# A Rapid Screening Strategy for Secondary Bacteriophages to Counter Phage Resistance in Carbapenem-Resistant *Acinetobacter baumannii*

**DOI:** 10.3390/antibiotics15090864

**Published:** 2026-09-04

**Authors:** Ruei-Sen Jiang, Li-Kuang Chen, Chun-Chieh Tseng

**Affiliations:** 1 Department and Graduate Institute of Public Health, Tzu Chi University, Hualien 97004, Taiwan; 111324103@gms.tcu.edu.tw; 2 Institute of Medical Sciences, College of Medicine, Tzu Chi University, Hualien 97004, Taiwan; lkc@tzuchi.com.tw; 3 Department of Clinical Pathology, Buddhist Tzu Chi General Hospital, Hualien 97002, Taiwan

**Keywords:** *Acinetobacter baumannii*, phage cocktail, phage resistance, secondary phage, next evolutionary phage typing

## Abstract

**Background**: Carbapenem-resistant *Acinetobacter baumannii* (CRAB) is a major nosocomial pathogen; bacteriophage therapy is a promising alternative, but its principal challenge is the rapid emergence of phage resistance. Cocktails counter this yet they are usually assembled by simply pooling phages active against the bacterium, not designed against the resistant mutants that arise. **Methods**: Next evolutionary phage typing (NEPT) addresses this by inducing resistance to a primary phage and then selecting secondary phages that lyse the resulting resistant mutant. Because choosing effective secondary phages still relies on qualitative visual reading of plaques (size and clarity), this study aimed to identify a rapid quantitative criterion. **Results**: Using the clinical strain CRAB 43895 and primary phage ϕ8, we obtained 65 NEPT-derived secondary phages that lyse the ϕ8-resistant mutant (ϕ8R) and combined four quantitative indicators—relative bacterial growth, lytic capability, coverage rate, and plaque morphology—with binary logistic regression to predict effective inhibition by the ϕ8 + secondary-phage cocktail (96-h OD_600_ < 0.1). Of the four, only lytic capability (the titer after 3 h of co-culture) independently predicted effective inhibition (ROC AUC = 0.76; at ≥10^7^ plaque-forming units (PFU)/mL, sensitivity 0.83, specificity 0.69). **Conclusions**: Lytic capability thus provides a rapid (~8–12 h), quantitative criterion for selecting effective secondary phages within the NEPT framework, improving on conventional qualitative typing.

## 1. Introduction

*Acinetobacter baumannii* is one of the most important nosocomial pathogens worldwide, causing pneumonia, bacteremia, and urinary tract and wound infections, and is notable for its ability to persist on inanimate surfaces and to rapidly accumulate antibiotic resistance [1,2]. Driven by the overuse of antibiotics, carbapenem-resistant *A. baumannii* (CRAB) has caused severe outbreaks in intensive care units worldwide, and the World Health Organization has designated it a critical-priority pathogen for which new antibacterial strategies are urgently needed [3].

Although colistin, a last-line agent against CRAB, retains partial activity, its nephrotoxicity and neurotoxicity limit clinical use [4,5], making non-antibiotic antibacterial approaches an urgent public-health need. Bacteriophages (phages) are viruses that specifically infect and lyse bacteria; their high host specificity, sparing of the normal microbiota, and abundance in the environment have renewed interest in them as alternatives or adjuvants to antibiotics [6,7]. We previously demonstrated the potential of phage ΦAB2 as an environmental biocontrol agent against multidrug-resistant *A. baumannii* [8].

However, when a single phage is used, bacteria readily develop resistance through the loss or modification of surface receptors that prevent phage adsorption, rapidly generating phage-resistant mutants [9,10]. To overcome this limitation, phage cocktails combining two or more phages are widely advocated to broaden the host range and delay the development of resistance [11,12].

In this study, phage typing refers to assessing a bacterial isolate’s susceptibility to a panel of lytic phages to identify suitable phages for therapeutic or decontamination applications, rather than to the conventional use of phage typing for epidemiological differentiation of bacterial isolates. Nevertheless, the phage typing currently used in clinical and environmental phage-spray decontamination relies on time-consuming visual, qualitative reading (approximately 8–12 h) that determines only whether a phage can lyse a given strain and cannot quantify the temporal dynamics of resistance [13,14]; moreover, evolutionary studies have shown that the timing and order of phage exposure markedly influence the trajectory of bacterial resistance evolution [15], yet there remains no systematic, quantitative basis for selecting which secondary (backup) phage to deploy once bacteria become resistant to the primary phage. We have previously found that antibiotic-resistant *A. baumannii* is more susceptible to environmental phages than antibiotic-sensitive strains [13], and have applied pre-optimized phage cocktails as aerosols for CRAB decontamination in intensive care and extracorporeal membrane oxygenation units, introducing next evolutionary phage typing (NEPT) to anticipate alternative phages effective after resistance emerges [14,16]; clinically, pre-optimized phage cocktails have also rapidly controlled secondary CRAB infection in critically ill COVID-19 patients [17].

Building on this, the present study used the clinical strain CRAB 43895 as an indicator, selected its primary phage ϕ8, and induced a phage-resistant mutant (ϕ8R); secondary phages capable of lysing ϕ8R were then identified by NEPT. By integrating four indicators—relative bacterial growth, lytic capability, coverage rate, and plaque morphology—with logistic regression analysis, we established a rapid and objective quantitative method for screening effective secondary phages, aiming to provide a practical basis for designing phage cocktails for clinical environmental decontamination.

## 2. Results

### 2.1. Phage Typing, NEPT, and Generation of Phage-Resistant Mutants

In the 2021 phage-typing surveillance of clinical CRAB, 308 CRAB isolates were classified into nine types according to their susceptibility to lytic phages, with the most prevalent type accounting for 60% (185/308). CRAB 43895 belonged to this dominant type and was therefore selected as the representative indicator strain.

Primary phage typing (PPT) of CRAB 43895 identified 31 lytic primary phages. After resistance to each primary phage was induced individually and the mutants were re-typed by NEPT, partial turbidity appeared as early as 6 h of co-culture (OD_600_ = 0.284), with confirmed resistance and active proliferation by 9 h (OD_600_ = 0.726); the loss of phage susceptibility was confirmed by the absence of plaque formation in the spot test. The longest time to resistance was 33 h (ϕRB99) (Table 1). Because cultures were assessed only at predefined time points, these values represent the earliest time point at which resistance was detected, rather than the exact time of resistance emergence. Accordingly, they were used only to categorize phages according to the relative speed of resistance development (fast or slow), rather than as quantitative estimates of emergence time. Nine primary phages (ϕ6, ϕR30, ϕR32–37, and ϕR51, all short-tailed podoviruses) failed to generate resistant mutants, whereas the remaining 22 successfully yielded resistant mutants.

Plaques were classified by size and clarity as L, G, C, O, or S (Figure S1); S and O, which indicated weak lysis, were excluded, and only L, G, and C were retained as candidate cocktail phages. Podovirus phages reached at best an L morphology, whereas myovirus (long contractile-tailed) phages reached at best a C morphology.

**Figure 1:**
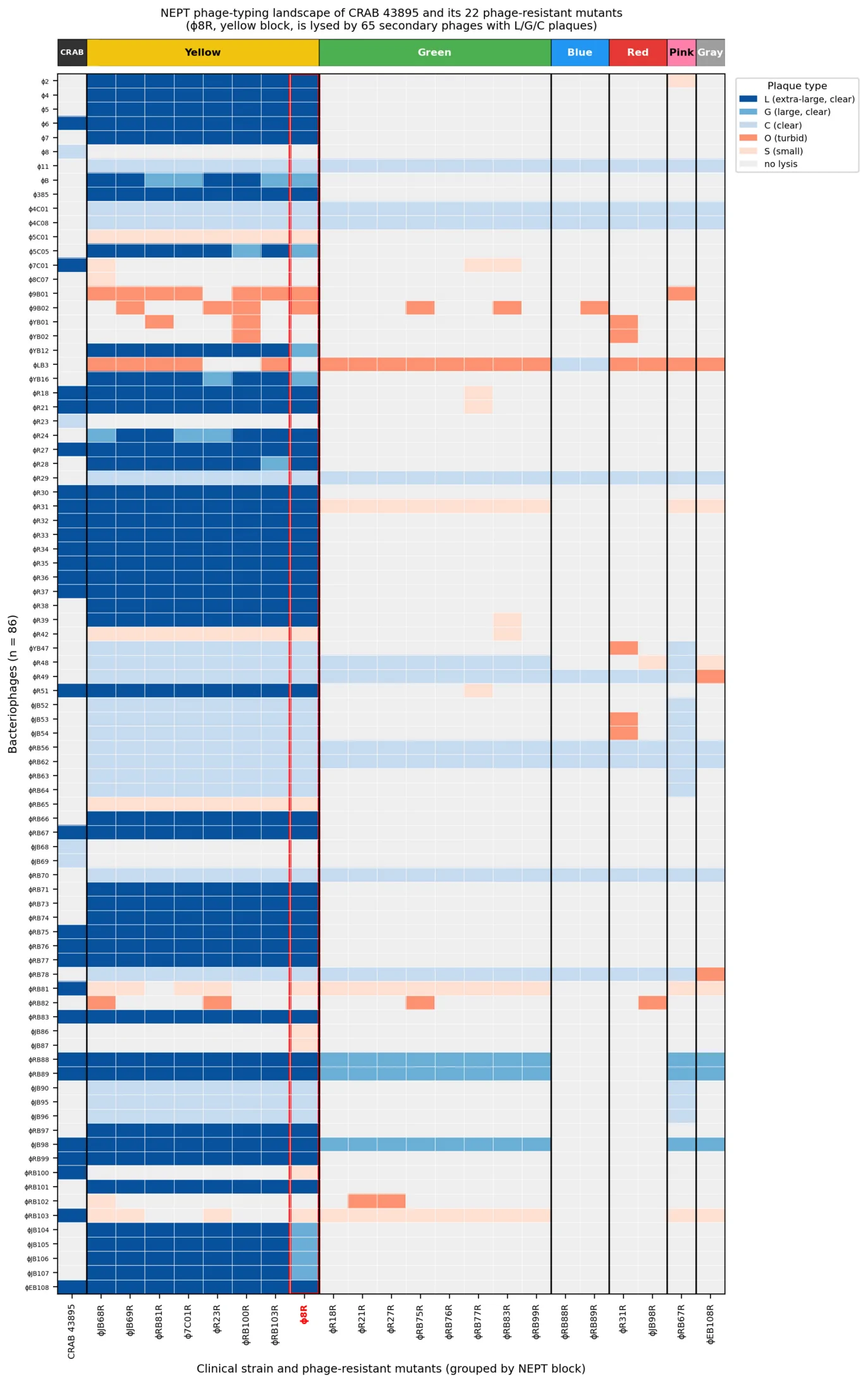
Next evolutionary phage typing (NEPT) landscape of CRAB 43895 and its 22 phage-resistant mutants. Of a laboratory collection of 144 *A. baumannii* phages screened, the 86 shown here lysed CRAB 43895 and/or at least one of its phage-resistant mutants. Each row is one of these 86 phages and each column is a bacterial strain—the parental clinical strain CRAB 43895 (leftmost column) and the 22 phage-resistant mutants grouped into six NEPT blocks (yellow, green, blue, red, pink, and gray). Cell color denotes the plaque phenotype of each phage on each strain, graded as L, G, C, S, O, or no lysis (see Figure S1 for the classification criteria). CRAB 43895 was lysed by 31 primary phages, whereas its ϕ8-resistant mutant ϕ8R (yellow block) remained susceptible to 65 secondary phages producing L, G, or C plaques. The red dashed box and red column label indicate ϕ8R, the phage-resistant mutant used for secondary-phage screening in this study.

By NEPT, the resistant mutants were grouped into six blocks according to their patterns of phage susceptibility (Figure 1). Among them, ϕ8R—derived from the primary myovirus phage ϕ8—displayed the broadest secondary-phage susceptibility, being lysed by the largest number of secondary phages, with the number of lytic phages increasing from the original 31 to 65. ϕ8R and its 65 secondary phages were therefore used for the subsequent four-indicator evaluation. ϕ8 was selected as the primary phage prior to resistance induction because it exhibited the highest adsorption efficiency among the 31 primary phages. Resistance to ϕ8 subsequently emerged within 9 h, and ϕ8 alone did not suppress CRAB 43895 over 96 h. Although these findings were not used to select ϕ8, they support its use as a model for evaluating whether secondary phages can control rapidly emerging phage resistance. After NEPT, ϕ8R was selected from the 22 resistant mutants because its broad susceptibility to secondary phages yielded the largest number of combinations for model construction.

### 2.2. Adsorption Efficiency of Primary Phages and Selection of ϕ8

**Figure 2:**
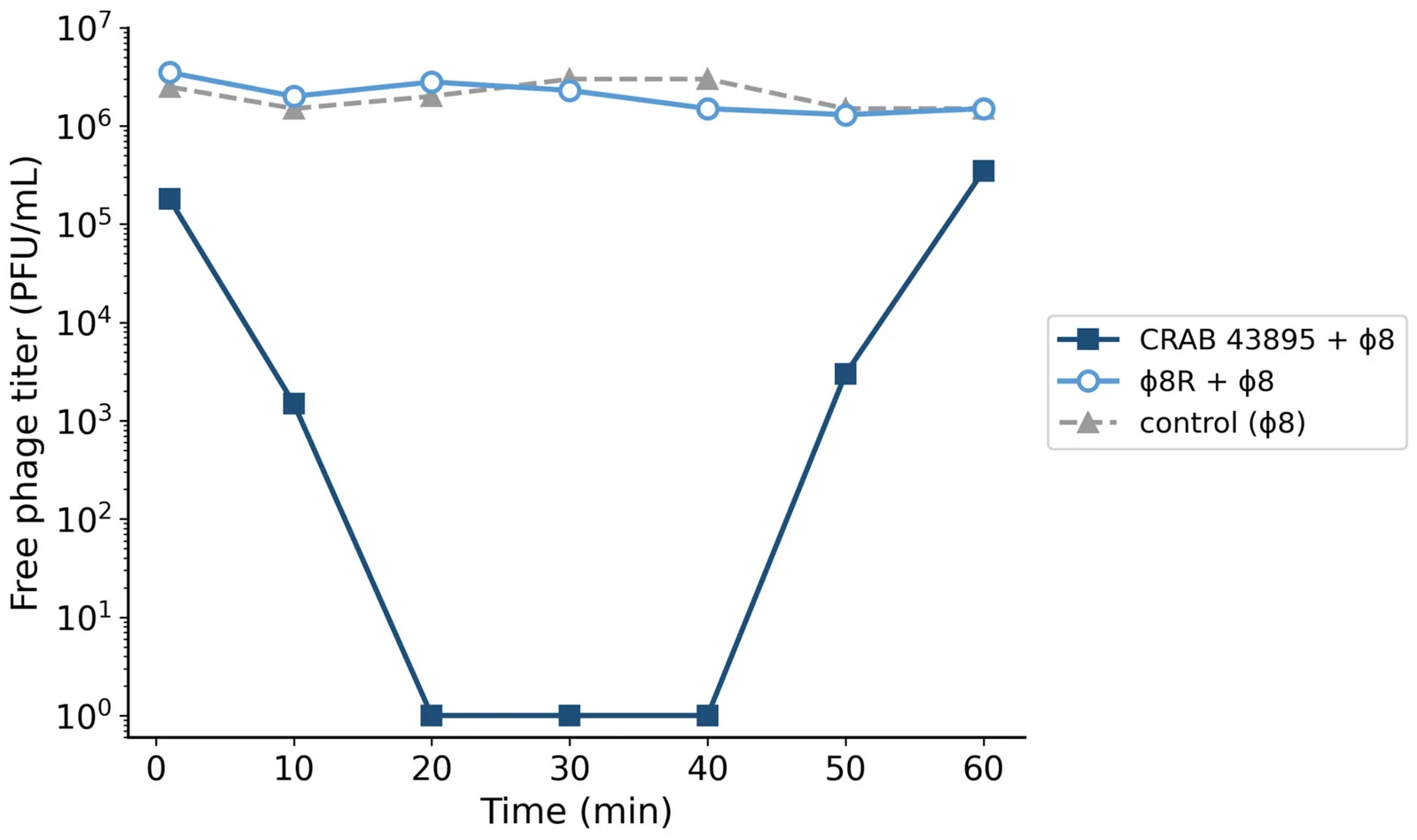
Adsorption of the primary phage ϕ8 to CRAB 43895 and its phage-resistant mutant ϕ8R. Free-phage titer (PFU/mL, log scale) was measured over 60 min for ϕ8 incubated with the clinical strain CRAB 43895 (dark blue squares) or the ϕ8-resistant mutant ϕ8R (light blue circles), together with a phage-only control (gray dashed line). With CRAB 43895, free ϕ8 became undetectable between 20 and 40 min (below the counting limit of an undiluted 2-µL spot, 500 PFU/mL), indicating near-complete adsorption to the host, before rebounding as progeny phages were released; with ϕ8R, the titer overlapped that of the control, indicating loss of adsorption. Points represent the mean of three replicates.

To provide a criterion for selecting the primary phage of the cocktail, the 31 phages lytic to CRAB 43895 were subjected to adsorption assays. Most primary phages showed a marked decrease in free-phage titer as early as 1 min, with ϕJB98 and ϕEB108 exhibiting the greatest reductions (98.8% and 99.9%, respectively); podovirus phages (ϕ6, ϕ7C01, ϕR18, ϕR21, ϕR27, ϕR30–37, ϕR51, ϕRB67, ϕRB75–77, ϕRB81, ϕRB83, ϕRB88–89, ϕRB99–100, and ϕRB103) decreased by 97.9–99.9% within 0–10 min. Myovirus phages (ϕ8, ϕR23, and ϕJB68–69) adsorbed more slowly but also decreased by 99.9% within 0–20 min; in particular, ϕ8 became undetectable between 20 and 40 min (a reduction of at least 99.95%, the titer falling below the counting limit of an undiluted 2-µL spot, 500 PFU/mL), indicating near-complete adsorption to the host and the highest adsorption efficiency, and it was therefore selected as the primary phage for subsequent experiments (Figure 2). The adsorption curves of the remaining 30 primary phages—each against CRAB 43895 and, where obtained, its corresponding phage-resistant mutant—are provided in Figure S2.

In contrast, the adsorption curves of the phage-resistant mutants (ϕ8R and the others) overlapped with those of the phage-only controls, indicating that once CRAB 43895 acquired resistance the phages could no longer adsorb, presumably owing to alterations in bacterial surface receptors (Figure 2 and Figure S2).

### 2.3. Growth Kinetics of CRAB 43895 and Its Phage-Resistant Mutant ϕ8R

**Figure 3:**
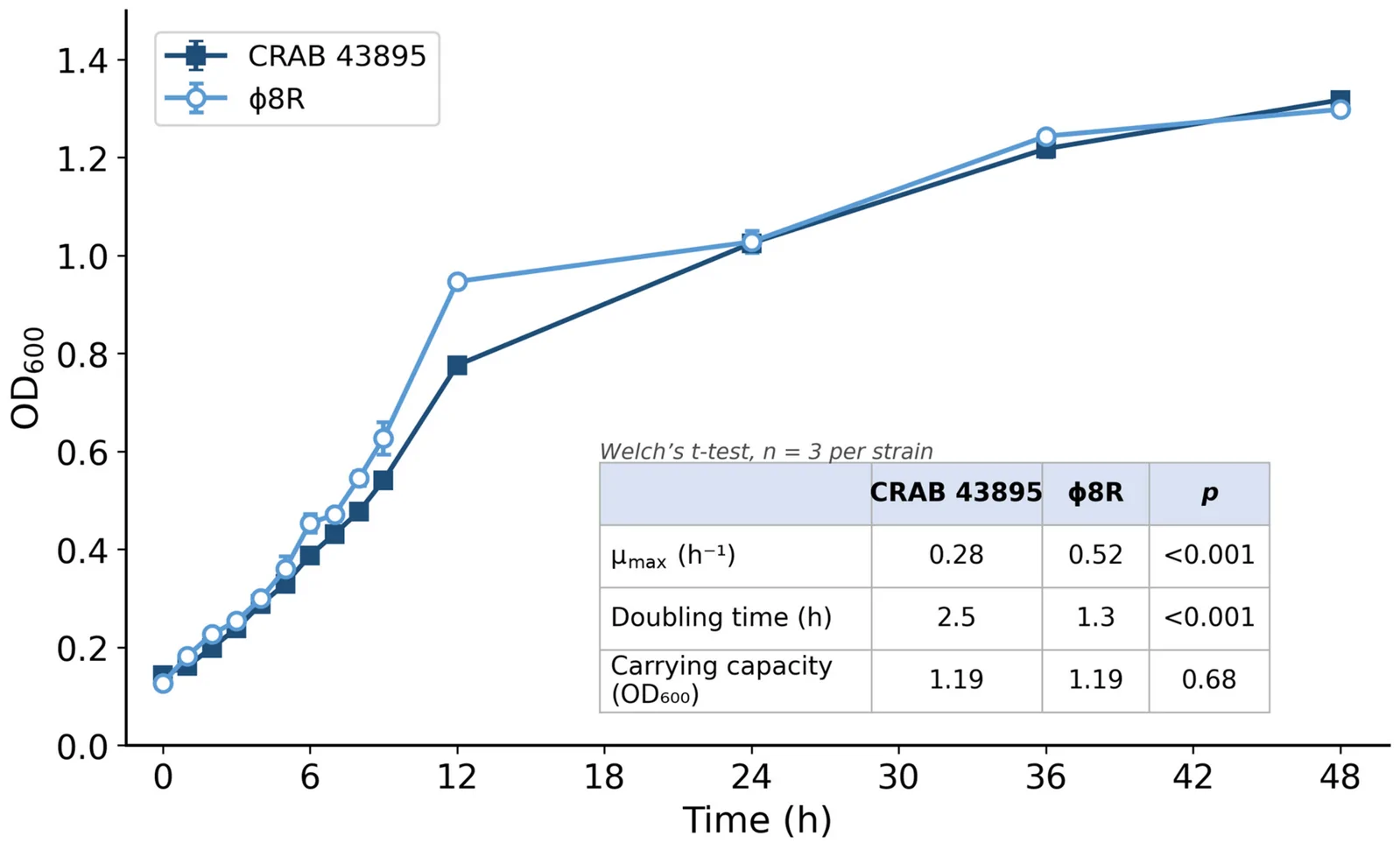
Growth curves of the clinical strain CRAB 43895 (dark blue squares) and its ϕ8-resistant mutant ϕ8R (light blue circles). OD_600_ was monitored over 48 h from a common starting density (OD_600_ = 0.1); points and error bars represent the mean ± SD of three replicates. The table within the figure (lower right) reports the maximum specific growth rate (μ_max_), doubling time, and carrying capacity for each strain (Welch’s *t*-test, *n* = 3). ϕ8R reached the same carrying capacity as the parental strain and grew at least as rapidly, indicating no growth-related fitness cost under these conditions.

To determine whether acquiring resistance to ϕ8 imposed a growth (fitness) cost, the growth of CRAB 43895 and its phage-resistant mutant ϕ8R was compared from a common starting density of OD_600_ = 0.1 (Figure 3). Each growth curve was summarized by two standard parameters—the maximum specific growth rate (μ_max_) during the exponential phase and the carrying capacity (the plateau OD_600_ reached at 24–48 h)—which were compared between strains (*n* = 3). During the exponential phase, ϕ8R grew at least as rapidly as the parental strain; its μ_max_ was in fact slightly higher (0.521 ± 0.014 vs. 0.281 ± 0.010 h^−1^, corresponding to doubling times of 1.3 and 2.5 h; Welch’s *t*-test, *p* < 0.001), consistent with the steeper rise in OD_600_ between 6 and 12 h. The two strains then converged to an indistinguishable carrying capacity (OD_600_ = 1.190 ± 0.011 vs. 1.187 ± 0.003, *p* = 0.68), and both reached 10^8^ CFU/mL at OD_600_ = 0.7 under identical conditions. Thus, resistance to ϕ8 did not impair the growth of CRAB 43895—if anything, ϕ8R grew marginally faster early in the exponential phase while reaching the same final density—indicating no growth-related fitness cost under the conditions tested. Other fitness-associated traits were not evaluated. This supports the use of ϕ8R as a robust host in the subsequent secondary-phage assays.

### 2.4. Coverage of the Phage-Resistant Mutants by Secondary Phages

The coverage of the panel of phage-resistant mutants by each secondary phage was evaluated and graded into level 1 (coverage ≥ 70%) or level 2 (<70%). Thirteen secondary phages were classified as level 1, of which ten were myoviruses and only three were podoviruses, whereas the remaining secondary phages were graded level 2 (full membership lists are given in Table S1). Thus, the secondary phages with the broadest coverage of the phage-resistant mutants were predominantly myoviruses, indicating an advantage of this morphotype in providing broad coverage against ϕ8R.

### 2.5. Relative Resistance of ϕ8R to the Secondary Phages

**Figure 4:**
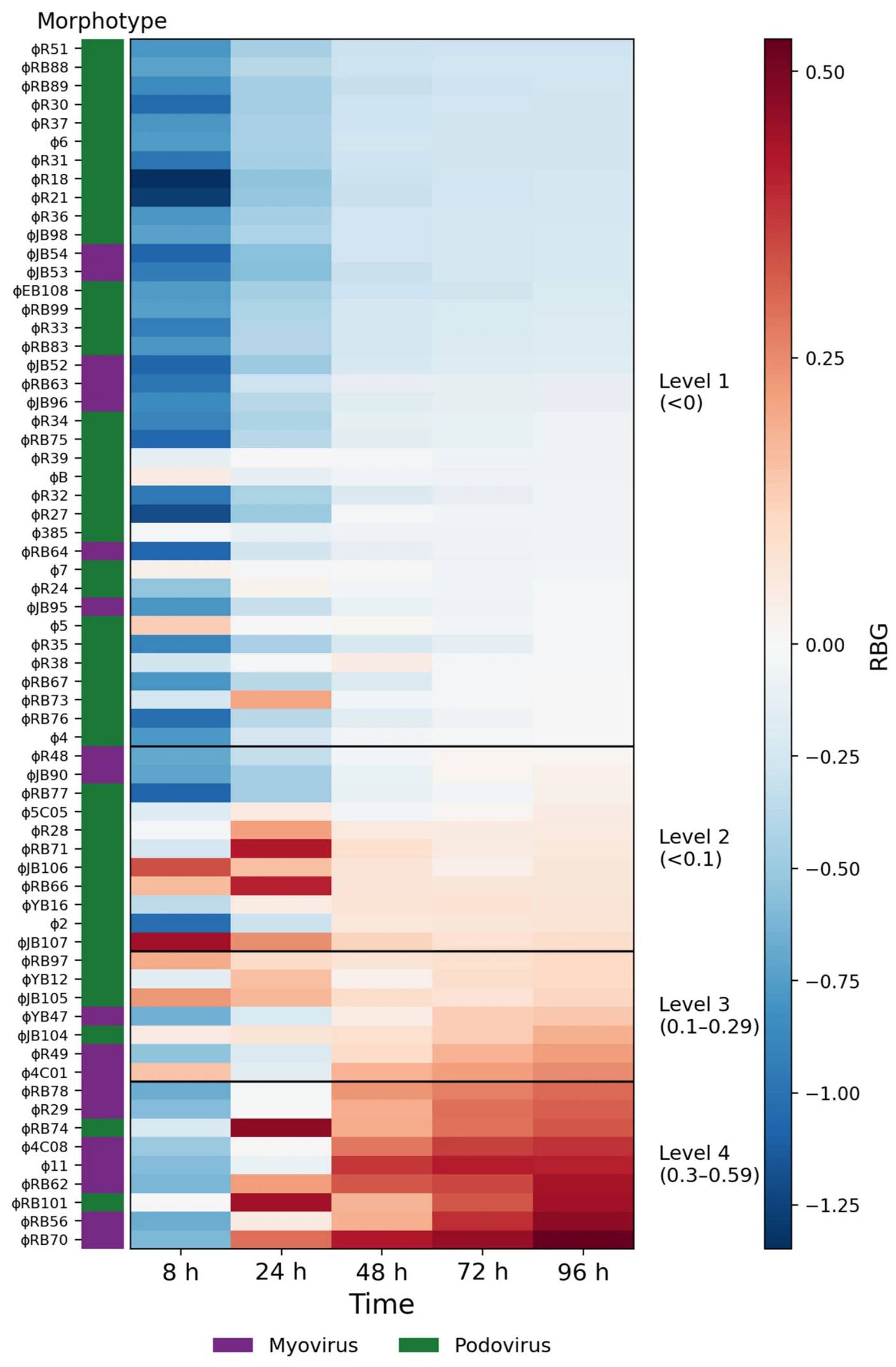
Relative bacterial growth (RBG) of the phage-resistant mutant ϕ8R co-cultured with each secondary phage. Each row is one of the 65 secondary phages (ordered by the 96-h RBG value); columns are the assay time points (8–96 h), and cell colour encodes RBG on a diverging scale centred at zero (blue, negative, indicating suppression of ϕ8R; red, positive, indicating regrowth). The left strip denotes phage morphotype (myovirus, podovirus). At the right, phages are grouped into levels 1–4 by their 96-h RBG (level 1, negative; level 2, <0.1; level 3, 0.1–0.29; level 4, 0.3–0.59); lower levels indicate stronger long-term suppression of ϕ8R.

Using ϕ8R as the indicator strain, the relative bacterial growth (RBG) of ϕ8R in the presence of each of the 65 secondary phages was monitored from 8 to 96 h (Figure 4); a lower RBG indicates stronger suppression of ϕ8R, whereas a higher value indicates a greater tendency of ϕ8R to regrow. Most secondary phages suppressed ϕ8R as early as 8 h (negative RBG), but for some the RBG rose progressively over time and suppression weakened, indicating that the inhibitory performance can only be judged over a prolonged period of up to 96 h. Based on the 96-h RBG value, the 65 phages were graded from level 1 (negative; best) to level 4 (0.3–0.59; worst); the secondary phages with the weakest suppression (level 4) were predominantly myoviruses (full grading in Table S2).

### 2.6. Lytic Capability of the Secondary Phages Against ϕ8R

**Figure 5:**
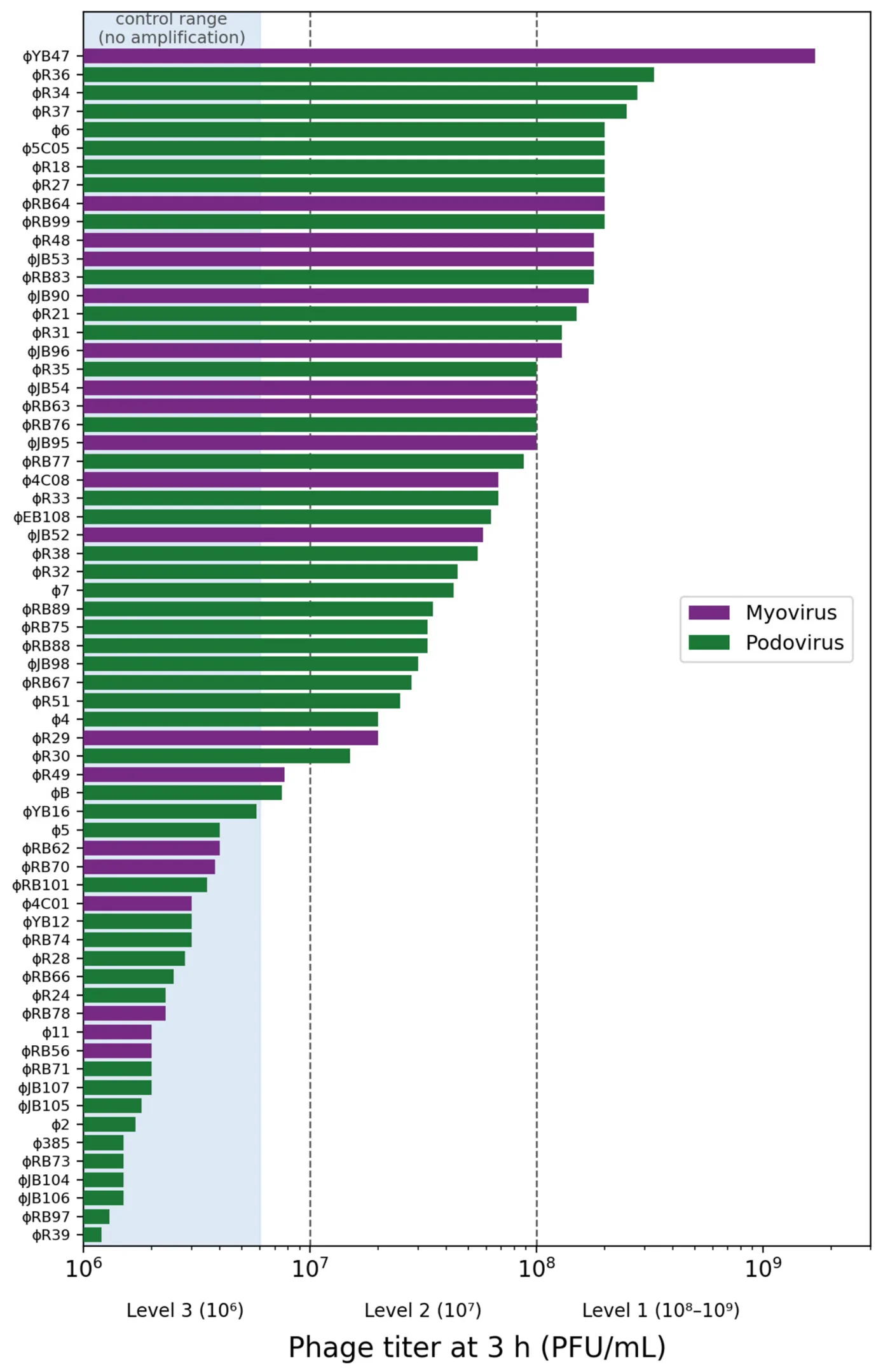
Lytic capability of the secondary phages against ϕ8R. Each horizontal bar is the phage titer (PFU/mL, log scale) of one of the 65 secondary phages after 3 h of co-culture with ϕ8R, ordered from highest to lowest; bar colour denotes phage morphotype (myovirus, podovirus). The shaded band marks the range of the phage-only controls (no amplification), and the dashed lines at 10^7^ and 10^8^ delimit the grading levels (level 1, 10^8^–10^9^; level 2, 10^7^; level 3, 10^6^).

The lytic (amplification) capability of the 65 secondary phages against ϕ8R was assessed by measuring the phage titer after 3 h of co-culture and comparing it with a phage-only control (Figure 5). Based on the 3-h titer, phages were graded as level 1 (10^8^–10^9^ PFU/mL), level 2 (10^7^), or level 3 (10^6^, equal to the control, i.e., no amplification). Most secondary phages amplified effectively (22 at level 1 and 17 at level 2), whereas 26 remained at the control level (level 3). Lytic capability showed no clear association with phage morphotype, with both myoviruses and podoviruses distributed across all three levels (full grading in Table S3).

### 2.7. Inhibition of the Clinical Strain by Individual Primary Phages

**Figure 6:**
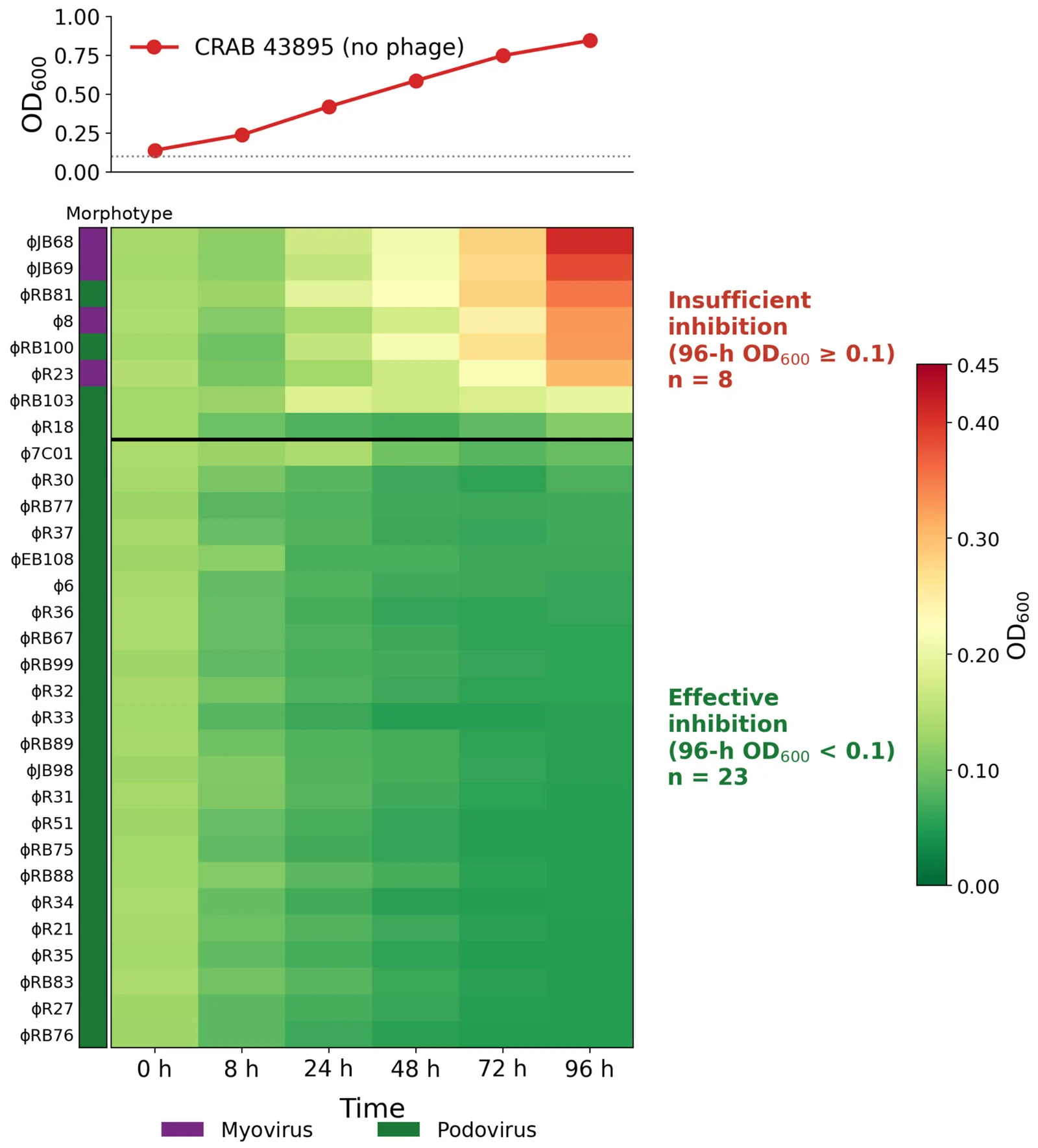
Inhibition of the clinical strain CRAB 43895 by individual primary phages. (Top) OD_600_ of CRAB 43895 without phage (no-phage control). (Heatmap) OD_600_ of CRAB 43895 co-cultured with each of the 31 primary phages over 0–96 h; rows are ordered by the 96-h OD_600_ and cell colour encodes OD_600_ (scale at right; dashed line, 0.1). The left strip indicates phage morphotype. Phages are grouped by 96-h OD_600_ into effective (<0.1) and insufficient (≥0.1) inhibition.

The inhibitory activity of each of the 31 primary phages against CRAB 43895 was assessed individually over 0–96 h (Figure 6). Using a 96-h OD_600_ < 0.1 as the criterion for effective inhibition, 23 phages were effective and 8 were insufficient. The eight insufficient phages comprised all four myovirus primary phages (ϕ8, ϕR23, ϕJB68, and ϕJB69) and four podovirus phages (ϕR18, ϕRB81, ϕRB100, and ϕRB103); all eight belonged to the group against which resistance emerged most rapidly (9 h).

### 2.8. Inhibition of the Clinical Strain by Phage Cocktails

**Figure 7:**
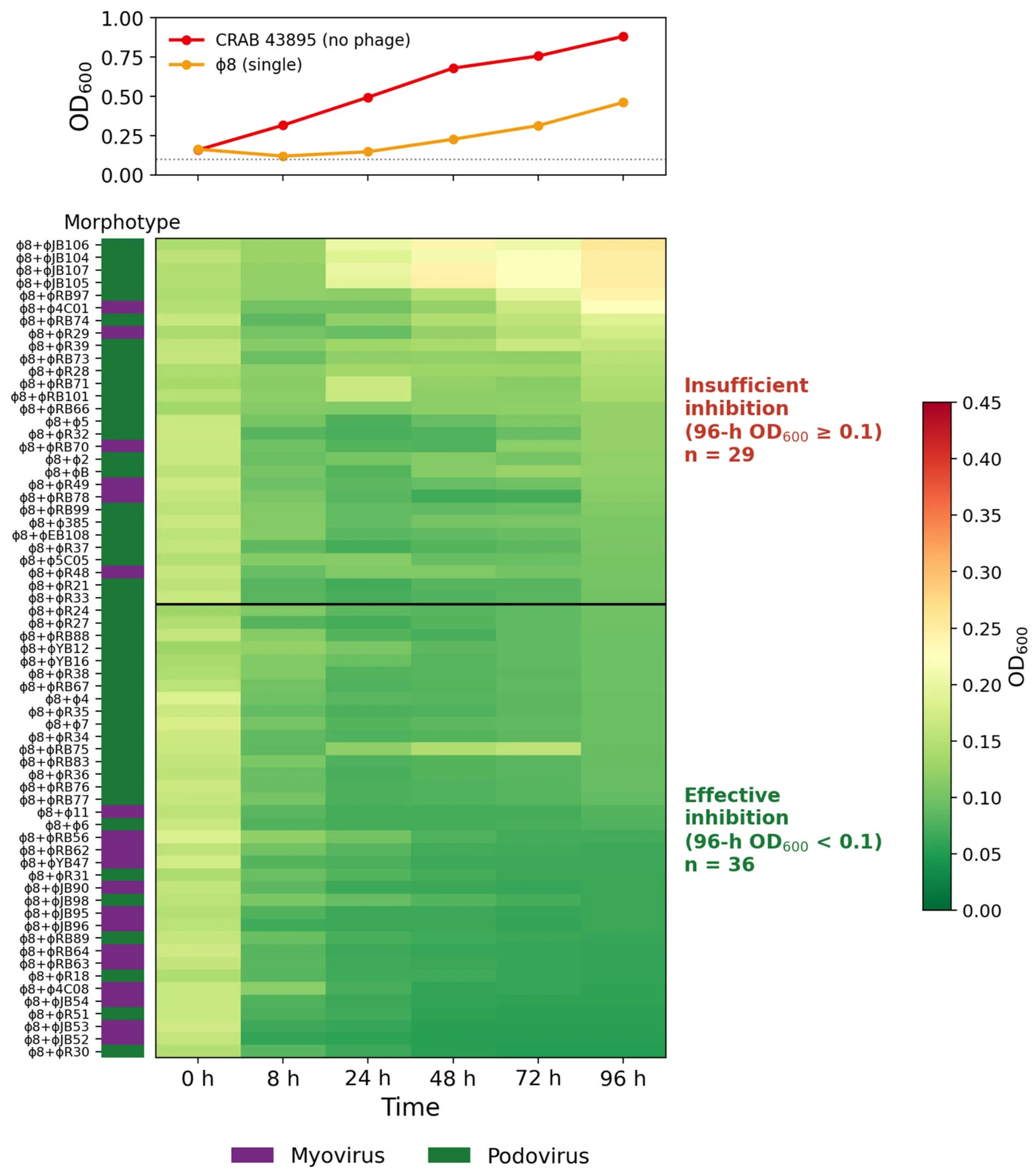
Inhibition of CRAB 43895 by the single primary phage ϕ8 versus ϕ8-containing cocktails. (Top) OD_600_ of the no-phage control and of single ϕ8. (Heatmap) OD_600_ of CRAB 43895 co-cultured with each ϕ8 + secondary-phage cocktail over 0–96 h; rows are ordered by the 96-h OD_600_ and the colour scale is identical to Figure 6 (dashed line, 0.1). The left strip indicates the morphotype of the secondary phage. Cocktails are grouped by 96-h OD_600_ into effective (<0.1, *n* = 36) and insufficient (≥0.1, *n* = 29) inhibition.

The inhibition of CRAB 43895 by the single primary phage ϕ8 was then compared with that of cocktails of ϕ8 plus each secondary phage (Figure 7). At 24 h only a few cocktails were less inhibitory than ϕ8 alone, but as incubation continued almost all cocktails outperformed the single phage by 72–96 h. Applying the same criterion (96-h OD_600_ < 0.1), 36 of the 65 cocktails achieved effective inhibition and 29 were insufficient. Overall, even after CRAB 43895 became resistant to the primary phage ϕ8, the secondary phage in the cocktail continued to suppress the bacterium, so the cocktails sustained inhibition over time more effectively than the single phage.

### 2.9. Descriptive Statistics and Logistic Regression of the Four Indicators

The four indicators of the 65 secondary phages—relative bacterial growth (RBG), lytic capability, coverage rate, and plaque morphology—were analysed against the dependent variable (effective inhibition by the ϕ8 cocktail, defined as a 96-h OD_600_ < 0.1). By the chi-square or Fisher’s exact test, lytic capability (*p* < 0.0001) and RBG (*p* = 0.019) were significantly associated with effective inhibition, whereas plaque morphology (*p* = 0.125) and coverage rate (*p* = 1.000) were not (Table 2). The measured values for each phage are listed in Table S4.

In the multivariable logistic regression, only lytic capability remained statistically significant after adjustment for the other indicators: relative to level 3 (titer equal to the control, i.e., no amplification), level 1 (10^8^–10^9^ PFU/mL) had an odds ratio (OR) of 11.74 (95% confidence interval (CI) 2.17–63.56, *p* = 0.004) and level 2 (10^7^) an OR of 9.09 (95% CI 1.53–54.06, *p* = 0.015), whereas RBG, plaque morphology, and coverage rate were non-significant (Table 3). The full model output is given in Table S5. Thus, a secondary phage that releases roughly ten-fold or more progeny than the control (≥10^7^ PFU/mL) is predictive of effective inhibition of CRAB 43895 when combined with ϕ8. Collinearity among the four indicators was low: GVIF ranged from 1.48 to 2.96, and the scaled GVIF^1/(2df)^ ranged from 1.16 to 1.32, with a maximum variance inflation factor of 4.25 for any single indicator level. These values provided no evidence of problematic multicollinearity among the predictors.

**Figure 8:**
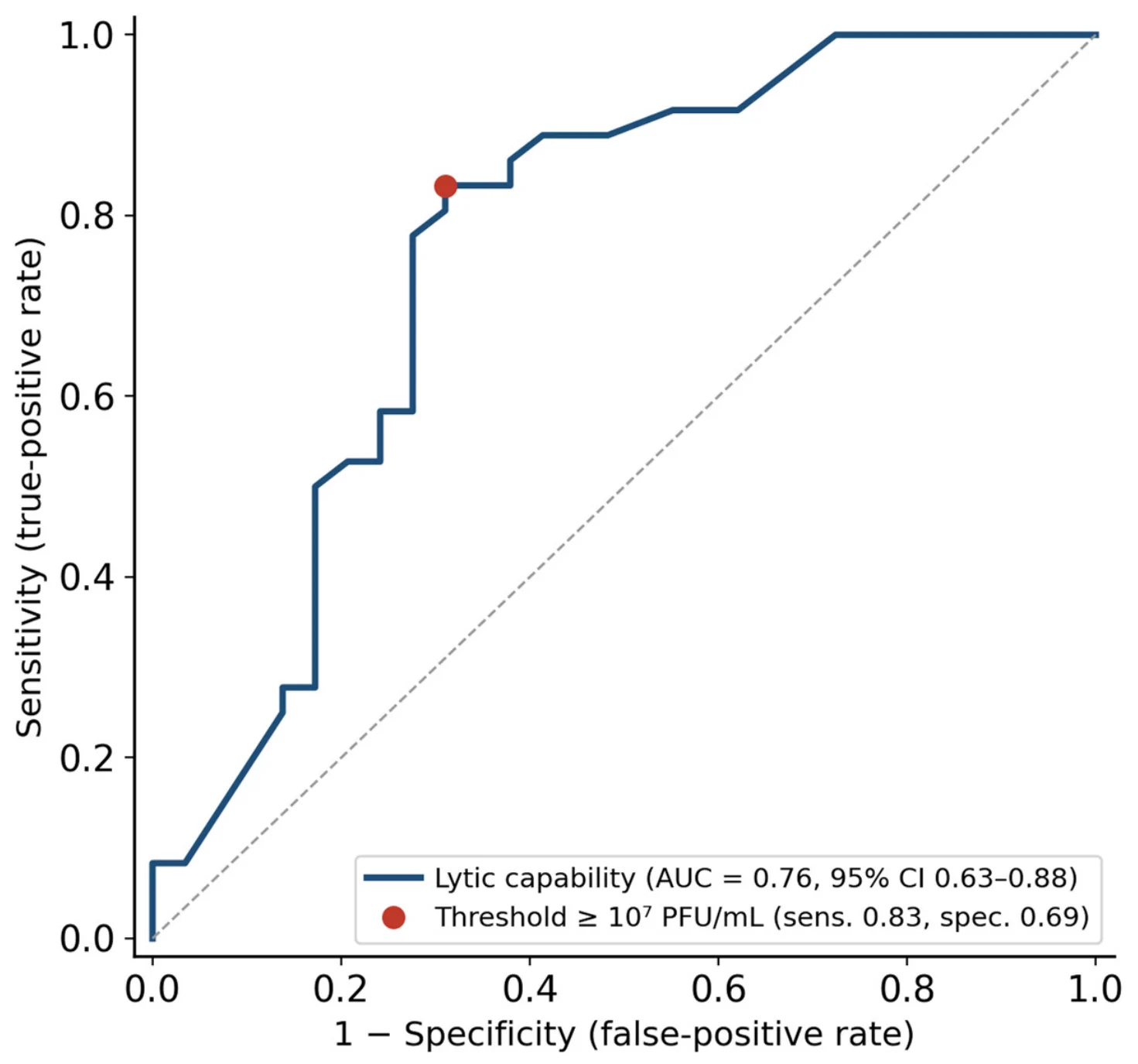
Receiver-operating-characteristic (ROC) curve for lytic capability as a predictor of effective cocktail inhibition. The 3-h lytic titer of each secondary phage was used to predict effective inhibition (96-h OD_600_ < 0.1) by the corresponding ϕ8 cocktail (*n* = 65). The area under the curve (AUC) was 0.76 (95% CI 0.63–0.88); the red point marks the operating threshold of a 3-h titer ≥ 10^7^ PFU/mL (sensitivity 0.83, specificity 0.69; positive and negative predictive values both 0.77). The dashed diagonal denotes random classification.

The influence of the outcome definition was examined by repeating the regression at 96-h OD_600_ thresholds ranging from 0.08 to 0.20 (Table S6). Between 0.08 and 0.12, lytic capability remained the only significant predictor (*p* ≤ 0.017), and the other three indicators remained non-significant. At 0.15 and above, the two outcome groups became strongly unbalanced (55:10 and 59:6), and no indicator reached significance. No threshold produced a model in which an indicator other than lytic capability was significant. As a screening test, lytic capability predicted effective inhibition with an area under the receiver-operating-characteristic curve (AUC) of 0.76 (95% CI 0.63–0.88); at a 3-h titer threshold of ≥10^7^ PFU/mL the sensitivity was 83% and the specificity 69% (Figure 8). At this threshold, the positive predictive value was 77% (30 of 39), and the negative predictive value was 77% (20 of 26). Because the lytic-capability assay is completed within 8–12 h, this indicator provides a practical basis for the rapid screening of effective secondary phages.

### 2.10. Confirmatory Experiments

To determine whether the inhibition observed with the cocktails required both phages or could be produced by the secondary phage alone, six representative secondary phages were retested against CRAB 43895 with a secondary-phage-alone treatment included (Section 4.10, Table S7). The six fell into two groups. For ϕR18, ϕR32, and ϕJB98, which also lyse CRAB 43895, the secondary phage alone was already sufficient: the 96-h OD_600_ was 0.062–0.066 and viable counts fell below 500 CFU/mL, closely matching the corresponding cocktails. For these phages, the assay cannot separate the contribution of the combination from that of the secondary phage. The single-phage treatment had already reduced the culture close to the medium blank (OD_600_ 0.049), leaving no range in which an additional effect could be resolved. For ϕJB53, ϕ4C01, and ϕJB106, which lyse only ϕ8R, the secondary phage alone did not approach the effectiveness criterion (96-h OD_600_ 0.924–1.072; 8.0 × 10^8^–1.3 × 10^9^ CFU/mL, against 1.263 and 2.0 × 10^9^ CFU/mL for the untreated control), and ϕ8 alone reached 0.544 and 4.2 × 10^8^ CFU/mL. Neither phage alone effectively inhibited CRAB 43895 in these three groups. The cocktails improved bacterial suppression, but only the combination of ϕ8 and ϕJB53 reduced the 96-h OD_600_ below 0.1 and was therefore classified as effective.

Viable counts measured in parallel supported the 96-h OD_600_ criterion. Among the treatments included in the confirmatory experiment, every treatment with a 96-h OD_600_ below 0.1 (0.062–0.079) had a viable count below 500 CFU/mL, whereas every treatment at or above 0.1 (0.135–1.263) retained 3.5 × 10^6^ to 2.0 × 10^9^ CFU/mL. The two groups did not overlap. The lowest viable count among treatments with OD_600_ ≥ 0.1 was more than three orders of magnitude above the <500 CFU/mL reporting threshold. Treatments with no detectable viable bacteria retained OD_600_ values slightly above the medium blank, supporting the use of <0.1 rather than the blank value as the inhibition threshold.

In the original experiment, the cocktail treatment received half the total phage dose that the single-phage treatment received. To make the comparison more balanced, the six cocktails were repeated with the same nominal total phage dose as the single-phage treatment. Increasing the total nominal MOI from 5 to 10 had little effect on five of the six cocktails. The OD_600_ of the ϕJB106 cocktail decreased from 0.260 to 0.135, but it remained above 0.1 and was still classified as insufficient. Equalizing the total nominal MOI did not change the classification of any of these six cocktails.

Nine treatments could be compared between the original and confirmatory experiments. Seven had the same classification in both experiments. The two different results were for ϕR18 alone and the ϕR32 cocktail. For ϕR18 alone, the mean 96-h OD_600_ decreased from 0.114 in the original experiment to 0.066 in the confirmatory experiment. The original mean exceeded the 0.1 threshold because of a single high replicate (0.232); the other two replicates were 0.059 and 0.051. For the ϕR32 cocktail, the mean decreased from 0.124 to 0.067. These were the two original values closest to the 0.1 threshold. All seven values farther from the threshold retained their original classifications. Treating ϕR32 as effective in the regression analysis did not change the result. Lytic capability remained the only significant indicator (*p* = 0.005). The ϕR18 result did not affect the model because the model did not include single-phage treatments. We retained the original classifications because the original and confirmatory experiments were independent.

## 3. Discussion

In the present study, we applied NEPT to the clinical strain CRAB 43895 and its primary phage ϕ8, obtained 65 secondary phages capable of lysing the resistant mutant ϕ8R, and, by combining four quantitative indicators with logistic regression, found that among the four indicators only lytic capability predicted which secondary phage would effectively inhibit the bacterium when used in a cocktail—an assay that can be completed within 8–12 h.

NEPT and conventional phage typing can qualitatively identify candidate phages that remain active after resistance emerges, but they cannot indicate which of these candidates will actually be effective; in previous applications of pre-optimized phage cocktails for environmental decontamination and treatment [14,16,17], the selection of secondary phages still relied on qualitative visual reading of plaques and lacked an objective, quantitative means of ranking them. The present study addresses this gap by providing a quantitative and rapid indicator that predicts the actual efficacy of each secondary phage before it is combined.

The adsorption assay showed that resistance of ϕ8R to ϕ8 was associated with loss of adsorption to the host surface. Loss or alteration of the ϕ8 receptor is the most likely explanation [18], but the receptor was not characterized in this study. Growth-curve analysis further showed that this resistance carried no growth-related fitness cost under the conditions tested, as ϕ8R reached the same carrying capacity as the parental strain CRAB 43895 and even grew slightly faster during the exponential phase. Because the resistant mutant showed no growth disadvantage under these conditions, it would not be counter-selected on the basis of growth rate alone, although other fitness components were not examined. This explains why reliance on a single primary phage is insufficient for durable control and underscores the need for a secondary phage that can still infect and suppress strains that have already become resistant to the primary phage.

That lytic capability was the only indicator independently predictive of effective inhibition points to amplification on ϕ8R as the relevant property: higher 3-h titers measured after co-culture with ϕ8R were associated with effective rather than insufficient inhibition in the 96-h cocktail assay. The intervening mechanisms, including changes in the phage-to-bacterium ratio and lytic pressure over time, were not measured directly.

The stability of this finding was examined by bootstrap resampling and repeated cross-validation. For lytic capability alone, the optimism-corrected and cross-validated AUCs were 0.74 and 0.72, close to the apparent value of 0.76. For the model containing all four indicators, the values were 0.73 and 0.68, both below the apparent value of 0.81. The single indicator was therefore the more stable basis for prediction.

This interpretation is consistent with observations in other systems. Alexyuk et al. reported that *Escherichia coli* phages with higher lytic activity inhibited host growth more durably [19], and Li et al. showed that the effectiveness of a phage cocktail depends not on the number of phage types but on their lytic efficiency [20]. The present study translates this principle into a quantitative and rapid screening criterion: lytic capability is determined from the phage titer after a 3-h co-culture, with a result available within about 8–12 h—comparable in turnaround to conventional phage typing but quantitative rather than relying on the qualitative visual reading of plaque size and clarity.

The same reasoning applies to the primary phage. Our study used adsorption efficiency as the sole criterion for selection, and it is not sufficient on its own. Adsorption is a necessary first step of infection, but it does not predict productive killing. ϕ8 was more completely adsorbed than any other primary phage, yet it did not suppress CRAB 43895 over 96 h (Figure 6). Burst size and latent period were not measured separately here. The 3-h titer is influenced by both and provides a composite measure of short-term amplification, which may explain why lytic capability predicted effective inhibition whereas adsorption did not. In terms of genome-based criteria, features such as receptor-binding proteins and the absence of lysogeny-associated genes would add a further layer to primary-phage selection.

Notably, although large, clear plaques are traditionally regarded as a hallmark of a superior phage, plaque morphology was not an independent predictor of inhibition. Plaque size and clarity are governed largely by adsorption rate and lysis timing rather than by the magnitude of phage amplification [21], so they need not track a phage’s capacity to suppress a resistant host, and selection based on plaque appearance alone may be misleading [22]. Whether this finding extends to other phage–host systems cannot be established from a single model. In the present dataset, plaque morphology was strongly skewed, with 57 of the 65 secondary phages in level 1 and only 8 in level 2, which limits the power to detect an effect. Testing in systems with more variable plaque morphology would be needed to confirm this.

The remaining two indicators were similarly uninformative once lytic capability was taken into account, though for different reasons. Relative bacterial growth (RBG) was associated with effective inhibition in the univariable analysis but lost significance after adjustment, because the long-term suppression it reflects is largely a downstream consequence of amplification and is therefore moderately associated with lytic capability—a phage that amplifies efficiently also tends to suppress the host over time. Coverage rate, in contrast, measures the breadth of resistant mutants a phage can infect rather than the potency with which it suppresses any one of them; because host range and productive killing are distinct phage properties [23], a broad host range does not guarantee effective inhibition of the specific target ϕ8R.

The relationship between phage morphotype and efficacy was likewise not straightforward. Myoviruses achieved the broadest coverage of resistant mutants yet were over-represented among the phages with the weakest long-term suppression, and among phages whose 3-h titer did not rise appreciably, most myoviruses failed to suppress ϕ8R whereas most podoviruses still did. This dissociation may reflect morphotype-related differences in amplification kinetics: some podoviruses may amplify more slowly, so that the 3-h titer underestimates their eventual lytic output even though they ultimately suppress the host, whereas myoviruses that have not amplified by 3 h tend to fail outright. This also suggests that the 3-h sampling point may be conservative for slowly amplifying phages and warrants further investigation; more importantly, it shows that neither morphotype nor coverage can substitute for a direct, quantitative measure of amplification when selecting secondary phages.

Cocktails of ϕ8 and a secondary phage suppressed CRAB 43895 more effectively than ϕ8 alone at the later time points (72 and 96 h). This is consistent with the established view that, by forcing the bacterium to counter several phages at once, phage cocktails delay the emergence of resistant mutants and sustain inhibition for longer [11,12,24,25].

The benefit of a cocktail, however, was not universal but depended on how rapidly resistance arose against the primary phage. In the experiment in which CRAB 43895 was induced to develop resistance to the first-line phages, resistance was first detected between 6 and 33 h; in the inhibition experiment, every single-phage failure occurred among the phages against which resistance arose most rapidly (first detected at 9 h). This group was not uniformly ineffective, however—7 of 15 still suppressed the host—whereas none of the phages whose resistance emerged later (≥22 h) failed, and phages that induced no detectable resistance suppressed the host durably, as expected. Rapid resistance emergence is therefore a necessary but not a sufficient condition for the failure of a single phage.

ϕ8 was one of the phages for which resistance was first detected at 9 h. This study therefore evaluated the secondary-phage screening strategy in a model of early primary-phage resistance. Notably, in this system the addition of a secondary phage to ϕ8 yielded no combination with poorer inhibition: although a few combinations were transiently inferior to the single phage at early time points (24–48 h), by 72 and 96 h no combination was inferior to ϕ8 alone.

The model was based on 65 secondary-phage combinations in the CRAB 43895–ϕ8 system. Lytic capability was the only significant predictor. Its performance changed little after bootstrap correction and cross-validation, indicating that the result was stable within this dataset. Further studies are needed to determine whether it also applies to other strain–phage systems. Because OD_600_ defines inhibition, the endpoint indicates suppression of bacterial growth rather than sterilization. These findings are stable within this dataset but remain exploratory until externally validated. The receptor used by ϕ8 was not identified, and the mechanism of resistance was inferred solely from adsorption data. Given the stochastic nature of resistance emergence and the single induction performed for each phage, the times in Table 1 were used for relative ranking rather than as quantitative estimates of resistance emergence rates.

This study is a proof of concept: in the CRAB 43895/ϕ8 model, it establishes the feasibility of screening effective secondary phages within 8–12 h using a single quantitative indicator—the 3-h lytic capability. As an in vitro study of a single clinical strain, a natural next step is to extend the strategy to additional clinical strains, different primary phages and other bacterial species, and to validate its predictive power in vivo and in real-world decontamination settings; combining it further with genomic and receptor analyses of the resistant mutants would deepen understanding of the morphotype-related differences and resistance mechanisms and help integrate this rapid, quantitative screening approach into NEPT-based decontamination and treatment workflows.

## 4. Materials and Methods

### 4.1. Bacterial Strains, Phages, and Culture Conditions

The carbapenem-resistant *Acinetobacter baumannii* strain CRAB 43895, a clinical isolate recovered from a patient sputum sample at Tzu Chi Hospital in 2021 under approved biosafety procedures, was used as the indicator strain. Bacteria were grown in LB medium (LB-Broth Lennox, LBX0102; Formedium Ltd., Swaffham, Norfolk, UK) at 37 °C; a single colony was inoculated into LB broth (37 °C, 100 rpm) and cultured to 10^8^ colony-forming units (CFU)/mL (OD_600_ = 0.7; Sunrise microplate absorbance reader, Tecan Austria GmbH, Grödig, Austria) before use, and stocks were preserved in 25% glycerol (BIONOVAS Biotechnology, Toronto, ON, Canada) at −80 °C. Bacterial density was estimated from OD_600_ for the preparation of working suspensions; viable counts were determined by serial dilution and plate counting where reported. The phages used were environmentally isolated *A. baumannii* phages capable of infecting CRAB 43895, belonging to the podovirus and myovirus morphotypes.

### 4.2. Phage Purification, Propagation, and Titer Determination

A single plaque was co-cultured with host bacteria (OD_600_ = 0.7) in LB broth (37 °C, 100 rpm) for 2–3 h, filtered through a 0.45-µm Acrodisc syringe filter with Supor membrane (catalogue no. 4614; Pall Corporation, Port Washington, NY, USA), and confirmed for lytic activity by spot test; propagation was performed at a multiplicity of infection (MOI) of 0.01–1, and filtrates were stored at 4 °C. Phage titers were determined by a double-layer agar plaque assay (0.75% top and 1.5% bottom LB agar), counting plaque-forming units (PFU) after overnight incubation at 37 °C. For the spot test, serially diluted phages prepared in a 96-well plate were spotted (2 µL) onto a bacterial lawn. All measurements were performed in triplicate.

### 4.3. Phage Typing and Next Evolutionary Phage Typing (NEPT)

For phage typing, 2 µL of each of 144 *A. baumannii* phages was spotted onto a double-layer agar lawn and incubated overnight at 37 °C; plaques were classified by size and clarity as L (transparent, diameter > 9 mm), G (transparent, diameter approximately 9 mm), C (transparent, diameter approximately 5–9 mm), O (nontransparent, regardless of size), or S (transparent, diameter < 5 mm), following the classification system established previously [26]. The primary phage was selected according to the phage typing results and the highest adsorption efficiency. For NEPT, a single primary phage was co-cultured with CRAB 43895 (100 µL phage plus 100 µL bacteria in 3 mL LB broth; 37 °C, 100 rpm) and compared hourly with a bacteria-only control; the culture was harvested once it shifted from clear to turbid, indicating resistance development. Single colonies were then re-isolated and re-typed to identify the secondary phages capable of lysing the resistant strain. Of the 144 phages screened, the 86 that lysed CRAB 43895 and/or at least one of its 22 phage-resistant mutants are shown in the NEPT landscape (Figure 1).

### 4.4. Adsorption Assay

Bacteria were grown to OD_600_ = 0.7 (~10^8^ CFU/mL) and incubated with phage at an MOI of 0.01 (phage, 10^6^ PFU/mL; bacteria, 10^8^ CFU/mL) in LB broth (30 °C, 100 rpm). Samples (500 µL) were collected at 1, 5, 10, 15, 20, 25, and 30 min for podoviruses and at 1, 10, 20, 30, 40, 50, and 60 min for myoviruses, filtered (0.45 µm), and the residual free-phage titer was measured by spot test to calculate adsorption efficiency; each time point was performed in triplicate. A parallel phage-only control without bacteria was run at every time point, and adsorption efficiency was calculated as (titer of the phage-only control − titer of the experimental culture) ÷ titer of the phage-only control × 100. Using the control rather than the initial titer as the denominator corrects for the natural loss of phage infectivity during incubation. Because progeny release causes the free-phage titer to rebound, only time points before the observed rebound were used to calculate adsorption efficiency. The sampling window was 30 min for podoviruses and 60 min for myoviruses, reflecting the slower adsorption of myoviruses observed in this system.

### 4.5. Relative Bacterial Growth (RBG)

RBG at each time point was calculated as follows [15], and the 96-h RBG value was used for grading (Section 4.11):

RBG*_ij_* = [OD_600_(*t*) − OD_600_(0 h)]*_ij_*/[OD_600_(*t*) − OD_600_(0 h)]_control,*j*_

where i denotes the phage and j denotes the bacterium.

### 4.6. Bacterial Growth Curve

To compare the growth of CRAB 43895 and its phage-resistant mutant ϕ8R, overnight cultures were diluted to OD_600_ = 0.1, and OD_600_ was measured with the same microplate absorbance reader at 0–9, 12, 24, 36, and 48 h in triplicate. Each replicate growth curve was characterized by its maximum specific growth rate (μ_max_), determined as the maximum slope of ln(OD_600_) during the exponential phase, and its carrying capacity, defined as the mean plateau OD_600_ at 24–48 h.

### 4.7. Coverage Rate

The infection breadth of each secondary phage against the panel of phage-resistant strains was calculated as:

Coverage rate (%) = (number of phage-resistant strains infected by a given secondary phage/total number of phage-resistant strains) × 100

### 4.8. Lytic Capability Assessment

To quantify progeny release by secondary phages against the phage-resistant strain ϕ8R, phage and bacteria were co-cultured at an MOI of 0.01 (phage, 10^6^ PFU/mL; bacteria, 10^8^ CFU/mL) in LB broth (37 °C, 100 rpm); samples (500 µL) were collected at 1, 2, and 3 h, filtered, and titrated by spot test in triplicate, with the 3-h titer representing lytic capability. Each phage was tested in three independent co-cultures. The counting limit of an undiluted 2-µL spot was 500 PFU/mL, because one plaque in 2 µL of undiluted sample corresponds to 500 PFU/mL; counts obtained from diluted spots were multiplied by the corresponding dilution factor. The lowest mean 3-h titer was 1.37 × 10^6^ PFU/mL, approximately 2740-fold above the counting limit. Replicate variability was low, with a median within-phage standard deviation of 0.034 log_10_ PFU/mL. All 195 measurements remained within the log-scale grade assigned to each phage. The titers were therefore used to classify the phages into three grades rather than as exact quantitative measurements.

### 4.9. Growth-Inhibition Assays with Phage Cocktails and the Primary Phage

Assays were performed in a final volume of 200 µL at 37 °C, with OD_600_ measured at 0, 8, 24, 48, 72, and 96 h in triplicate. Phage suspensions were standardized before use so that equal volumes delivered equal PFU doses. The cocktail group comprised 100 µL LB broth, 50 µL bacteria (10^6^ CFU/mL), 25 µL primary phage (5 × 10^6^ PFU/mL), and 25 µL secondary phage (5 × 10^6^ PFU/mL); the single-primary-phage group comprised 100 µL LB broth, 50 µL bacteria, and 50 µL primary phage (10^7^ PFU/mL); and the bacteria-only control comprised 150 µL LB broth and 50 µL bacteria. Each well received a nominal bacterial input of 5 × 10^4^ CFU. The cocktail group received a total of 2.5 × 10^5^ PFU, with each phage contributing 1.25 × 10^5^ PFU, giving a total nominal MOI of 5. The single-phage group received 5 × 10^5^ PFU, giving a nominal MOI of 10.

### 4.10. Confirmatory Experiments with Additional Controls and Viable Counts

Six secondary phages were selected for the confirmatory experiment. In the original cocktail screen, ϕR18, ϕJB98, and ϕJB53 were classified as effective, whereas ϕR32, ϕ4C01, and ϕJB106 were classified as insufficient. Among them, ϕR18, ϕR32, and ϕJB98 also lyse CRAB 43895, whereas ϕJB53, ϕ4C01, and ϕJB106 lyse only ϕ8R. Together, these phages represented both morphotypes and all three levels of lytic capability.

For each secondary phage, six treatments were tested in a final volume of 200 µL. Two treatments contained a cocktail of ϕ8 and the secondary phage at a total nominal MOI of 10 or 5. The total nominal MOI 5 treatment reproduced the original condition described in Section 4.9. The remaining treatments were the secondary phage alone at a nominal MOI of 10, ϕ8 alone at a nominal MOI of 10, an untreated bacterial control, and an LB broth blank.

Each cocktail well contained 100 µL of LB broth, 50 µL of bacterial suspension, 25 µL of ϕ8, and 25 µL of secondary phage. Each single-phage well contained 100 µL of LB broth, 50 µL of bacterial suspension, and 50 µL of phage. The untreated control contained 150 µL of LB broth and 50 µL of bacterial suspension. In contrast, the blank contained 200 µL of LB broth. Phage suspensions were adjusted to 1 × 10^7^ PFU/mL, except that each phage in the total nominal MOI 5 cocktail was adjusted to 5 × 10^6^ PFU/mL. Before the assay, bacterial density was estimated from OD_600_ and diluted to a nominal concentration of 1 × 10^6^ CFU/mL. Based on this estimate, each well received a nominal bacterial input of 5 × 10^4^ CFU, and phage-containing wells received either 5.0 × 10^5^ PFU at nominal MOI 10 or 2.5 × 10^5^ PFU at total nominal MOI 5.

Each treatment was tested in three independent wells on two parallel plates with identical layouts. One plate was used for OD_600_ measurements, and the other for viable count measurements. Cultures were incubated at 37 °C, and OD_600_ was measured at 0, 8, 24, 48, 72, and 96 h. A treatment was classified as effective when the mean OD_600_ of the three wells at 96 h was below 0.1.

Viable counts were measured on the parallel plate so that sample removal did not affect the OD_600_ measurements. Samples were collected immediately after mixing (0 h) and at 24 and 96 h. From each well, 20 µL was withdrawn and serially diluted ten-fold through 10^−6^. Two microlitres of the undiluted sample and each dilution were spotted on LB agar in technical triplicate. Colonies were counted after overnight incubation at 37 °C, and the three technical replicates were averaged to obtain a single CFU/mL value for each well. When no colonies were observed in any of the three undiluted spots, the one-sided 95% upper confidence limit was approximately 500 CFU/mL based on the total examined volume of 6 µL. These samples were therefore reported as <500 CFU/mL. The 0-h counts ranged from 2.5 × 10^6^ to 5.2 × 10^6^ CFU/mL, higher than expected from the nominal inoculum. This difference is consistent with the limited precision of OD-based estimation of bacterial density. The 0-h counts were nevertheless comparable across all treatment groups, confirming similar starting bacterial loads. The MOI values therefore describe the intended experimental inputs and not infection ratios recalculated from the 0-h viable counts. The results are presented in Table S7.

### 4.11. Statistical Analysis

Statistical analyses were performed using IBM SPSS Statistics v23 (IBM Corp., Armonk, NY, USA) and R v4.5.1 (R Foundation for Statistical Computing, Vienna, Austria). *p* < 0.05 was considered statistically significant. All tests were two-sided.

To compare the growth of CRAB 43895 and ϕ8R, each replicate growth curve was reduced to two parameters—the maximum specific growth rate (μ_max_) and the carrying capacity—which were then compared between strains by Welch’s *t*-test (*n* = 3).

The dependent variable Y was binarized from the 96-h mean OD_600_ of the 65 combinations (≥0.1 = 0, insufficient inhibition; <0.1 = 1, effective inhibition). Associations between the four indicators and the dependent variable were analyzed by the chi-square test or, where any expected count was below five, by Fisher’s exact test (Freeman–Halton extension for tables larger than 2 × 2), and their predictive value was assessed by binary logistic regression. Odds ratios are reported with 95% confidence intervals and Wald *p*-values. Before fitting the multivariable model, collinearity among the four indicators was assessed by variance inflation factors, using the generalized variance inflation factor (GVIF) and its scaled form GVIF^1/(2df)^ for the multi-level indicators.

The four independent variables were graded as follows: RBG (based on the 96-h value) into four levels [level 1, negative; level 2, <0.1; level 3, 0.1–0.29; level 4, 0.3–0.59]; lytic capability (3-h titer, PFU/mL) into three levels [level 1, 10^8^–10^9^; level 2, 10^7^; level 3, 10^6^ (equal to control)]; coverage rate into two levels [level 1, ≥70%; level 2, <70%]; and plaque morphology into two levels. Because the best attainable plaque morphology differed between phage morphotypes (podoviruses reaching L, whereas myoviruses reached only C), the best clear morphology observed within each morphotype—L for podoviruses and C for myoviruses—was assigned to level 1 and G to level 2. The reference category was level 4 for RBG, level 3 for lytic capability, and level 2 for coverage rate and plaque morphology.

The 70% cutoff for coverage rate was determined empirically from the observed distribution rather than from previous studies. Coverage values were bimodal: 52 phages infected 8–9 of the 22 resistant mutants (36.4–40.9%), and 13 infected 17–22 of the 22 resistant mutants (77.3–100%). The cutoff was placed within the gap separating the two groups. The 96-h OD_600_ cutoff of 0.1 was likewise defined with reference to the assay itself. It lies below the density of the starting inoculum (OD_600_ 0.124–0.209), so a culture below this value had not grown over the 96-h period. It is also about twice the sterile-medium blank (OD_600_ 0.047) and about one-tenth of the untreated control at 96 h (OD_600_ 0.882).

To test whether the choice of cutoff influenced the results, a sensitivity analysis was performed in which the outcome was redefined at 96-h OD_600_ thresholds from 0.08 to 0.20, and the logistic regression was refitted at each threshold (Table S6). Because the ordinary maximum-likelihood fit showed quasi-complete separation at several of these thresholds, the sensitivity analysis was carried out by Firth penalized likelihood, each indicator being tested as a whole by a penalized likelihood-ratio test.

The binary logistic regression model was:

ln[*P*(Y = 1)/(1 − *P*(Y = 1))] = β_0_ + β_1_*X*_1_ + β_2_*X*_2_ + β_3_*X*_3_ + β_4_*X*_4_

where X_1_ = RBG, X_2_ = lytic capability, X_3_ = coverage rate, and X_4_ = plaque morphology.

The discriminative performance of lytic capability for predicting effective inhibition was evaluated by receiver-operating-characteristic (ROC) analysis on the continuous log_10_-transformed 3-h titer (area under the curve with a 2000-sample bootstrap 95% confidence interval); the chi-square/Fisher and ROC analyses were carried out in R v4.5.1. Internal validation was performed using two approaches. Optimism-corrected AUC was estimated using Harrell’s bootstrap procedure with 1000 resamples. For each bootstrap sample, the model was refitted, and its AUC was calculated in both the bootstrap sample and the original dataset. The mean difference between these two AUCs was taken as the optimism and subtracted from the apparent AUC. Cross-validated AUC was estimated using stratified five-fold cross-validation repeated 200 times. In each split, the model was refitted using four folds and evaluated using the held-out fold. The cross-validated AUC was calculated as the mean AUC across all 1000 held-out folds. Both procedures were applied to the model containing lytic capability alone and to the model containing all four indicators, using the graded variables defined above. Sensitivity, specificity and predictive values were calculated at a titer of ≥10^7^ PFU/mL. Results of the confirmatory experiment are reported as the mean ± standard deviation of three independent wells; no inferential testing was applied.

## Figures and Tables

**Table 1:** Time to first detection of resistance in CRAB 43895 against each primary phage during co-culture.

Phage	3	5	6	9	11	22	24	25	27	28	29	33	48	Time to Resistance (h)
ϕ6 *	0	0	0	0	0	0	0	0	0	0	0	0	0	>48
ϕ8	0	0	0.5	1	-	-	-	-	-	-	-	-	-	9
ϕ7C01	0	0	0	0	0	0.5	0.5	1	-	-	-	-	-	25
ϕR18	0	0	0.5	1	-	-	-	-	-	-	-	-	-	9
ϕR21	0	0	0.5	1	-	-	-	-	-	-	-	-	-	9
ϕR23	0	0	0.5	1	-	-	-	-	-	-	-	-	-	9
ϕR27	0	0	0	0	0.5	1	-	-	-	-	-	-	-	22
ϕR30 *	0	0	0	0	0	0	0	0	0	0	0	0	0	>48
ϕR31	0	0	0	0	0	0.5	0.5	0.5	0.5	0.5	1	-	-	29
ϕR32 *	0	0	0	0	0	0	0	0	0	0	0	0	0	>48
ϕR33 *	0	0	0	0	0	0	0	0	0	0	0	0	0	>48
ϕR34 *	0	0	0	0	0	0	0	0	0	0	0	0	0	>48
ϕR35 *	0	0	0	0	0	0	0	0	0	0	0	0	0	>48
ϕR36 *	0	0	0	0	0	0	0	0	0	0	0	0	0	>48
ϕR37 *	0	0	0	0	0	0	0	0	0	0	0	0	0	>48
ϕR51 *	0	0	0	0	0	0	0	0	0	0	0	0	0	>48
ϕRB67	0	0	0.5	1	-	-	-	-	-	-	-	-	-	9
ϕJB68	0	0	0.5	1	-	-	-	-	-	-	-	-	-	9
ϕJB69	0	0	0.5	1	-	-	-	-	-	-	-	-	-	9
ϕRB75	0	0	0.5	1	-	-	-	-	-	-	-	-	-	9
ϕRB76	0	0	0.5	1	-	-	-	-	-	-	-	-	-	9
ϕRB77	0	0	0.5	1	-	-	-	-	-	-	-	-	-	9
ϕRB81	0	0	0.5	1	-	-	-	-	-	-	-	-	-	9
ϕRB83	0	0	0.5	1	-	-	-	-	-	-	-	-	-	9
ϕRB88	0	0	0	0	0.5	1	-	-	-	-	-	-	-	22
ϕRB89	0	0	0	0	0	0.5	0.5	0.5	0.5	0.5	1	-	-	29
ϕJB98	0	0	0	0	0	0.5	0.5	0.5	0.5	0.5	1	-	-	29
ϕRB99	0	0	0	0	0	0.5	0.5	0.5	0.5	0.5	0.5	1	-	33
ϕRB100	0	0	0.5	1	-	-	-	-	-	-	-	-	-	9
ϕRB103	0	0	0.5	1	-	-	-	-	-	-	-	-	-	9
ϕEB108	0	0	0.5	1	-	-	-	-	-	-	-	-	-	9

Note: 0, clear (no resistance); 0.5, partial turbidity; 1, turbid (resistance developed); -, not determined after resistance was confirmed. Cell color follows the same scale (pale yellow, 0; amber, 0.5; brown, 1). The last column lists the time to resistance; “>48” denotes primary phages against which CRAB 43895 did not develop resistance within 48 h (marked with *). Cultures were assessed only at the scheduled time points indicated above. Thus, each value represents the first time point at which resistance was detected. In contrast, the actual emergence of resistance occurred sometime between that time point and the preceding one. Each phage was used for a single induction experiment.

**Table 2:** Distribution of the four indicators of the 65 secondary phages and their association with effective cocktail inhibition.

Variable	N (%)	*p*-Value
Relative bacterial growth (RBG)		0.019 *
Level 1	38 (58.5)	
Level 2	11 (16.9)	
Level 3	7 (10.8)	
Level 4	9 (13.8)	
Lytic capability		<0.0001 *
Level 1	22 (33.8)	
Level 2	17 (26.2)	
Level 3	26 (40.0)	
Plaque morphology		0.125
Level 1	57 (87.7)	
Level 2	8 (12.3)	
Coverage rate		1.000
Level 1	13 (20.0)	
Level 2	52 (80.0)	
Cocktail inhibition (dependent variable)		
Insufficient	29 (44.6)	
Effective	36 (55.4)	

Note: N, number of phages. Associations were tested by the chi-square test (lytic capability) or Fisher’s exact test (RBG, plaque morphology, coverage rate); * *p* < 0.05.

**Table 3:** Univariable and multivariable logistic regression of the four indicators predicting effective cocktail inhibition.

Variable	Unadjusted OR (95% CI)	*p*-Value	Adjusted OR (95% CI)	*p*-Value
Relative bacterial growth (RBG)				
Level 4 (reference)	—	—	—	—
Level 3	0.50 (0.06, 4.09)	0.518	0.53 (0.04, 7.05)	0.633
Level 2	0.47 (0.07, 3.04)	0.427	0.26 (0.02, 3.65)	0.318
Level 1	3.07 (0.69, 13.61)	0.140	0.93 (0.09, 10.09)	0.950
Lytic capability				
Level 3 (reference)	—	—	—	—
Level 2	10.83 (2.55, 45.96)	0.001 *	9.09 (1.53, 54.06)	0.015 *
Level 1	11.33 (2.93, 43.78)	<0.0001 *	11.74 (2.17, 63.56)	0.004 *
Plaque morphology				
Level 2 (reference)	—	—	—	—
Level 1	4.44 (0.82, 23.93)	0.083	0.71 (0.08, 6.64)	0.760
Coverage rate				
Level 2 (reference)	—	—	—	—
Level 1	0.93 (0.27, 3.13)	0.901	1.45 (0.22, 9.64)	0.702

Note: OR, odds ratio; CI, confidence interval; —, not estimated (reference category). The dependent variable is effective inhibition (96-h OD_600_ < 0.1). * *p* < 0.05.

## Data Availability

The summarized data supporting the findings of this study are provided in the article and Supplementary Materials. The complete replicate-level dataset is available from the corresponding author upon reasonable request.

## Supplementary Materials

The following supporting information can be downloaded at: https://www.mdpi.com/article/10.3390/antibiotics15090864/s1, Figure S1: plaque morphology used to grade phage lytic activity. Representative spot-test plaques on double-layer agar were classified by size and clarity as L (transparent, diameter > 9 mm), G (transparent, diameter approximately 9 mm), C (transparent, diameter approximately 5–9 mm), S (transparent, diameter < 5 mm), and O (nontransparent, regardless of size), following the classification system established previously [26]. Only L, G, and C were retained as candidate cocktail phages; S and O, indicating weak lysis, were excluded; Figure S2: adsorption kinetics of the remaining 30 primary phages (ϕ8 is shown in Figure 2). For each phage, the free-phage titer (PFU/mL, log scale) is plotted over time during incubation with the clinical strain CRAB 43895 (dark blue squares) and, where a resistant mutant was obtained, with the corresponding phage-resistant mutant (light blue open circles), alongside a phage-only control (gray dashed triangles). Free phage incubated with CRAB 43895 dropped sharply as the phage adsorbed and then rebounded as progeny were released, whereas titers with the resistant mutants overlapped those of the controls, indicating loss of adsorption. The nine podovirus phages for which CRAB 43895 did not develop resistance (ϕ6, ϕR30, ϕR32–37, and ϕR51) are shown with the clinical strain and control only. Each time point was assayed in triplicate; Table S1: coverage of the phage-resistant mutants by the secondary phages, graded by coverage rate; Table S2: relative bacterial growth (RBG) of ϕ8R against each secondary phage, graded by the 96-h RBG value; Table S3: lytic capability of the secondary phages against ϕ8R, graded by the phage titer at 3 h; Table S4: measured values for each of the 65 secondary phages; Table S5: complete output of the multivariable logistic regression model; Table S6: sensitivity of the logistic regression model to the definition of effective inhibition; Table S7: confirmatory experiment: 96-h OD_600_ and viable counts for six representative secondary phages against the clinical strain CRAB 43895.
