# Supplementary material for: A Rapid Screening Strategy for Secondary Bacteriophages to Counter Phage Resistance in Carbapenem-Resistant Acinetobacter baumannii
Source: Antibiotics (Basel). 2026 Sep 4;15(9):864. doi: 10.3390/antibiotics15090864 (PMC13603275; doi:10.3390/antibiotics15090864)
Supplement: Supplementary file 1 [file antibiotics-15-00864-s001.zip › antibiotics-4470173-supplementary.pdf]

Supplementary Material

A Rapid Screening Strategy for Secondary Bacteriophages to Counter Phage Resistance in Carbapenem-Resistant *Acinetobacter baumannii*

Ruei-Sen Jiang [a,†], Li-Kuang Chen [b,c,†] and Chun-Chieh Tseng [a,*]

[a] Department and Graduate Institute of Public Health, Tzu Chi University, Hualien 97004, Taiwan; 111324103@gms.tcu.edu.tw (R.-S.J.); tsengcc@mail.tcu.edu.tw (C.-C.T.)

[b] Institute of Medical Sciences, College of Medicine, Tzu Chi University, Hualien 97004, Taiwan; lkc@tzuchi.com.tw

[c] Department of Clinical Pathology, Buddhist Tzu Chi General Hospital, Hualien 97002, Taiwan

† These authors contributed equally to this work.

* Correspondence: tsengcc@mail.tcu.edu.tw; Tel./Fax: +886-3-8574179

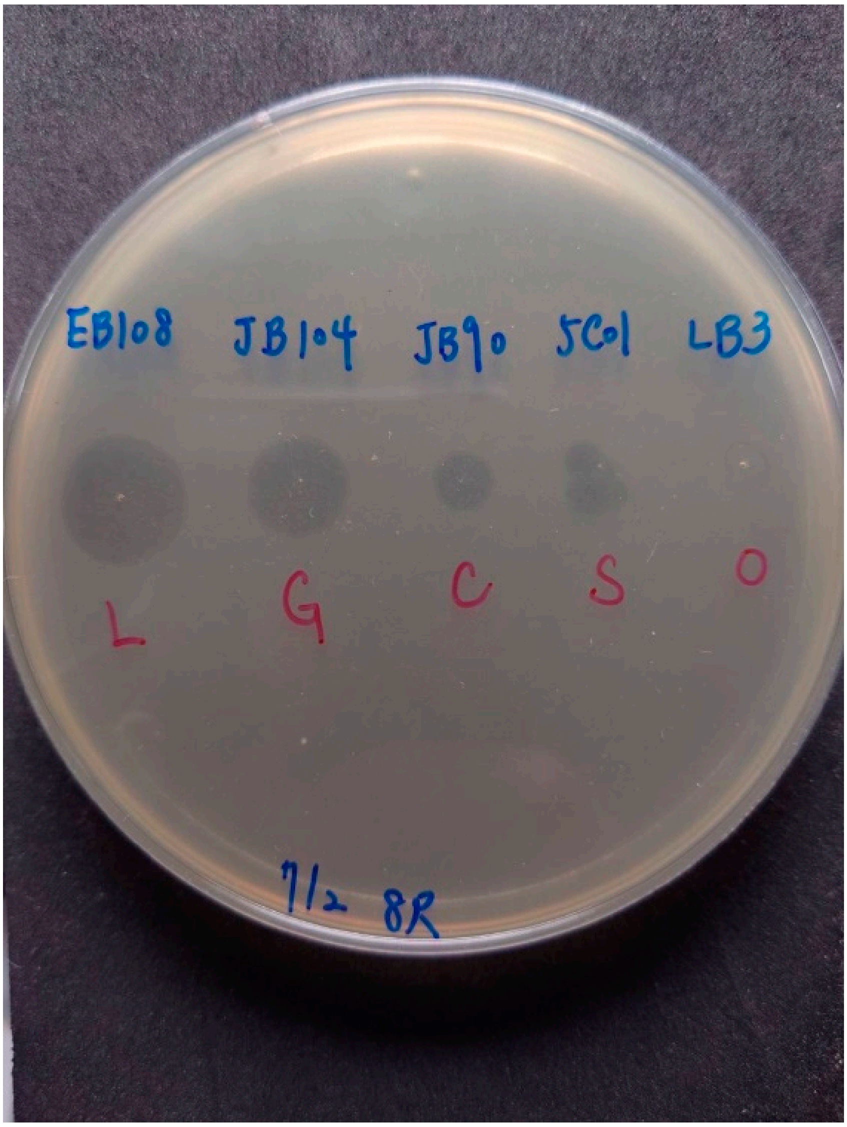

Figure S1. Plaque morphology used to grade phage lytic activity. Representative spot-test plaques on double-layer agar were classified by size and clarity as L (transparent, diameter > 9 mm), G (transparent, diameter approximately 9 mm), C (transparent, diameter approximately 5–9 mm), S (transparent, diameter < 5 mm), and O (nontransparent, regardless of size), following the classification system established previously [26]. Only L, G, and C were retained as candidate cocktail phages; S and O, indicating weak lysis, were excluded.

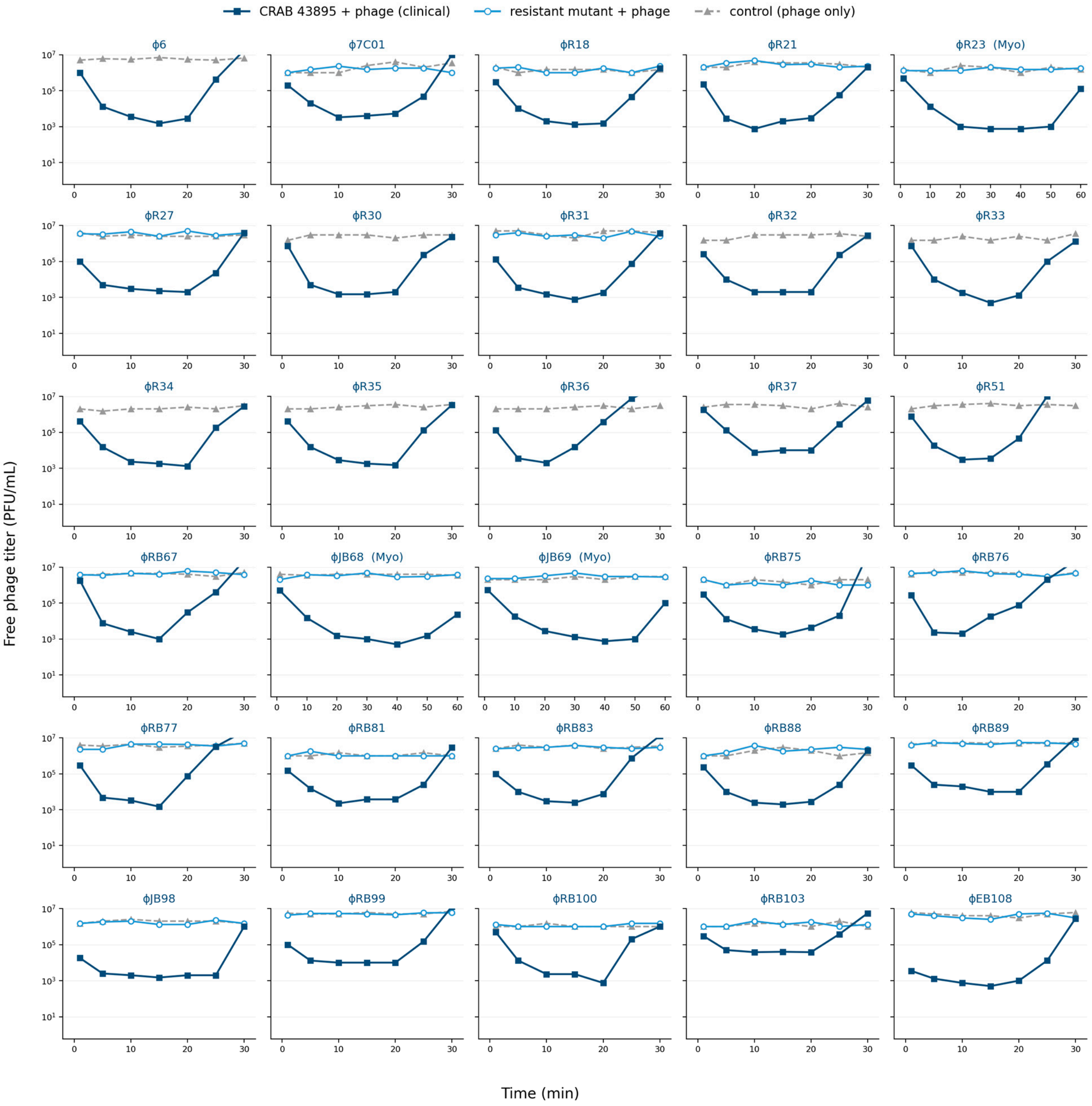

Figure S2. Adsorption kinetics of the remaining 30 primary phages (ϕ8 is shown in Figure 2). For each phage, the free-phage titer (PFU/mL, log scale) is plotted over time during incubation with the clinical strain CRAB 43895 (dark blue squares) and, where a resistant mutant was obtained, with the corresponding phage-resistant mutant (light blue open circles), alongside a phage-only control (gray dashed triangles). Free phage incubated with CRAB 43895 dropped sharply as the phage adsorbed and then rebounded as progeny were released, whereas titers with the resistant mutants overlapped those of the controls, indicating loss of adsorption. The nine podovirus phages for which CRAB 43895 did not develop resistance (ϕ6, ϕR30, ϕR32–37, and ϕR51) are shown with the clinical strain and control only. Each time point was assayed in triplicate.

Table S1. Coverage of the phage-resistant mutants by the secondary phages, graded by coverage rate.

Grade	Coverage rate	Myovirus	Podovirus
**Level 1**	≥70%	ϕ11, ϕ4C01, ϕ4C08, ϕR29, ϕR48, ϕR49, ϕRB56, ϕRB62, ϕRB70, ϕRB78	ϕRB88, ϕRB89, ϕJB98
**Level 2**	<70%	ϕYB47, ϕJB52–JB54, ϕRB63, ϕRB64, ϕJB90, ϕJB95, ϕJB96	ϕ2, ϕ4–7, ϕB, ϕ385, ϕ5C05, ϕYB12, ϕYB16, ϕR18, ϕR21, ϕR24, ϕR27, ϕR28, ϕR30–R39, ϕR51, ϕRB66, ϕRB67, ϕRB71, ϕRB73–RB77, ϕRB83, ϕRB97, ϕRB99, ϕRB101, ϕEB108, ϕJB104, ϕJB105, ϕJB106, ϕJB107

Note: the coverage rate is the proportion of the 22 phage-resistant mutants that a given secondary phage can infect; level 1, coverage ≥70%; level 2, <70%. Observed values were bimodal: level 1, 17–22 of the 22 mutants (77.3–100%); level 2, 8–9 of the 22 mutants (36.4–40.9%). Morphotype assignments follow the laboratory phage database.

Table S2. Relative bacterial growth (RBG) of ϕ8R against each secondary phage, graded by the 96-h RBG value.

Grade	RBG (96 h)	Myovirus	Podovirus
**Level 1**	Negative	ϕJB52, ϕJB53, ϕJB54, ϕJB95, ϕRB63, ϕRB64, ϕJB96	ϕ4, ϕ5, ϕ6, ϕ7, ϕ385, ϕB, ϕEB108, ϕJB98, ϕR18, ϕR21, ϕR24, ϕR27, ϕR30, ϕR31, ϕR32, ϕR33, ϕR34, ϕR35, ϕR36, ϕR37, ϕR38, ϕR39, ϕR51, ϕRB67, ϕRB73, ϕRB75, ϕRB76, ϕRB83, ϕRB88, ϕRB89, ϕRB99
**Level 2**	<0.1	ϕJB90, ϕR48	ϕ2, ϕ5C05, ϕR28, ϕRB66, ϕRB71, ϕRB77, ϕYB16, ϕJB106, ϕJB107
**Level 3**	0.1–0.29	ϕ4C01, ϕR49, ϕYB47	ϕRB97, ϕYB12, ϕJB104, ϕJB105
**Level 4**	0.3–0.59	ϕ4C08, ϕ11, ϕR29, ϕRB56, ϕRB62, ϕRB70, ϕRB78	ϕRB74, ϕRB101

Note: RBG was graded from the mean 96-h value into level 1 (negative), level 2 (<0.1), level 3 (0.1–0.29), and level 4 (0.3–0.59); lower grades indicate stronger long-term suppression of ϕ8R. Morphotype assignments follow the laboratory phage database.

Table S3. Lytic capability of the secondary phages against ϕ8R, graded by the phage titer at 3 h.

Grade	Titer at 3 h (PFU/mL)	Myovirus	Podovirus
**Level 1**	10^8–10^9	ϕJB53, ϕJB54, ϕJB90, ϕJB95, ϕR48, ϕRB63, ϕRB64, ϕYB47, ϕJB96	ϕ5C05, ϕ6, ϕR18, ϕR21, ϕR27, ϕR31, ϕR34, ϕR35, ϕR36, ϕR37, ϕRB76, ϕRB83, ϕRB99
**Level 2**	10^7	ϕ4C08, ϕJB52, ϕR29	ϕ4, ϕ7, ϕEB108, ϕJB98, ϕR30, ϕR32, ϕR33, ϕR38, ϕR51, ϕRB67, ϕRB75, ϕRB77, ϕRB88, ϕRB89
**Level 3**	10^6	ϕ4C01, ϕ11, ϕR49, ϕRB56, ϕRB62, ϕRB70, ϕRB78	ϕ2, ϕ5, ϕ385, ϕB, ϕR24, ϕR28, ϕR39, ϕRB66, ϕRB71, ϕRB73, ϕRB74, ϕRB97, ϕRB101, ϕYB12, ϕYB16, ϕJB104, ϕJB105, ϕJB106, ϕJB107

Note: lytic capability was graded from the 3-h phage titer into level 1 (10^8–10^9 PFU/mL), level 2 (10^7), and level 3 (10^6, equal to the phage-only control, i.e. no amplification). Morphotype assignments follow the laboratory phage database.

Table S4. Measured values for each of the 65 secondary phages.

Phage	Morphotype	3-h titer (PFU/mL), mean ± SD	Level	Coverage (%)	Level	RBG at 96 h, mean ± SD	Level	Plaque level	Cocktail 96-h OD_{600}, mean ± SD	Outcome
ϕ2	Podovirus	$(1.67 \pm 0.06) \times 10^6$	3	36.4	2	0.0741 ± 0.0375	2	1	0.121 ± 0.058	Insufficient
ϕR27	Podovirus	$(2.17 \pm 0.15) \times 10^8$	1	36.4	2	-0.0551 ± 0.2384	1	1	0.097 ± 0.006	Effective
ϕR51	Podovirus	$(2.50 \pm 0.30) \times 10^7$	2	36.4	2	-0.2743 ± 0.0157	1	1	0.056 ± 0.001	Effective
ϕRB77	Podovirus	$(8.60 \pm 0.26) \times 10^7$	2	36.4	2	0.0274 ± 0.1893	2	1	0.088 ± 0.001	Effective
ϕ4	Podovirus	$(2.03 \pm 0.25) \times 10^7$	2	36.4	2	-0.0017 ± 0.0730	1	1	0.095 ± 0.009	Effective
ϕR28	Podovirus	$(2.60 \pm 0.20) \times 10^6$	3	36.4	2	0.0519 ± 0.0472	2	1	0.148 ± 0.064	Insufficient
ϕJB52	Myovirus	$(5.53 \pm 0.31) \times 10^7$	2	40.9	2	-0.1614 ± 0.1226	1	1	0.053 ± 0.000	Effective
ϕRB78	Myovirus	$(2.17 \pm 0.15) \times 10^6$	3	95.5	1	0.2999 ± 0.1127	4	1	0.115 ± 0.079	Insufficient
ϕ5	Podovirus	$(3.97 \pm 0.25) \times 10^6$	3	36.4	2	-0.0251 ± 0.0127	1	1	0.124 ± 0.065	Insufficient
ϕR29	Myovirus	$(2.17 \pm 0.15) \times 10^7$	2	100.0	1	0.3160 ± 0.0933	4	1	0.173 ± 0.012	Insufficient
ϕJB53	Myovirus	$(1.63 \pm 0.15) \times 10^8$	1	40.9	2	-0.2108 ± 0.0242	1	1	0.055 ± 0.000	Effective
ϕRB83	Podovirus	$(1.63 \pm 0.15) \times 10^8$	1	36.4	2	-0.1777 ± 0.0712	1	1	0.092 ± 0.003	Effective
ϕ6	Podovirus	$(2.47 \pm 0.42) \times 10^8$	1	36.4	2	-0.2439 ± 0.0191	1	1	0.076 ± 0.014	Effective
ϕR30	Podovirus	$(1.40 \pm 0.17) \times 10^7$	2	36.4	2	-0.2497 ± 0.0342	1	1	0.052 ± 0.001	Effective
ϕJB54	Myovirus	$(1.13 \pm 0.23) \times 10^8$	1	40.9	2	-0.2155 ± 0.0339	1	1	0.059 ± 0.008	Effective
ϕRB88	Podovirus	$(3.37 \pm 0.12) \times 10^7$	2	81.8	1	-0.2638 ± 0.0181	1	1	0.096 ± 0.054	Effective
ϕ7	Podovirus	$(4.30 \pm 0.30) \times 10^7$	2	36.4	2	-0.0384 ± 0.0383	1	1	0.094 ± 0.009	Effective
ϕR31	Podovirus	$(1.37 \pm 0.21) \times 10^8$	1	36.4	2	-0.2432 ± 0.0182	1	1	0.065 ± 0.012	Effective
ϕRB56	Myovirus	$(2.13 \pm 0.15) \times 10^6$	3	100.0	1	0.4724 ± 0.0380	4	1	0.070 ± 0.021	Effective
ϕRB89	Podovirus	$(3.50 \pm 0.30) \times 10^7$	2	81.8	1	-0.2582 ± 0.0388	1	1	0.063 ± 0.004	Effective
ϕ11	Myovirus	$(2.30 \pm 0.30) \times 10^6$	3	100.0	1	0.4119 ± 0.1808	4	1	0.080 ± 0.028	Effective
ϕR32	Podovirus	$(4.43 \pm 0.12) \times 10^7$	2	36.4	2	-0.0552 ± 0.2006	1	1	0.124 ± 0.025	Insufficient
ϕRB62	Myovirus	$(4.20 \pm 0.20) \times 10^6$	3	100.0	1	0.4412 ± 0.0640	4	1	0.067 ± 0.004	Effective
ϕJB90	Myovirus	$(1.53 \pm 0.21) \times 10^8$	1	40.9	2	0.0217 ± 0.3671	2	1	0.064 ± 0.003	Effective
ϕB	Podovirus	$(7.53 \pm 0.35) \times 10^6$	3	36.4	2	-0.0635 ± 0.0605	1	2	0.120 ± 0.047	Insufficient
ϕR33	Podovirus	$(6.67 \pm 0.15) \times 10^7$	2	36.4	2	-0.1846 ± 0.0848	1	1	0.100 ± 0.015	Insufficient
ϕRB63	Myovirus	$(1.17 \pm 0.15) \times 10^8$	1	40.9	2	-0.1114 ± 0.2017	1	1	0.060 ± 0.007	Effective
ϕJB95	Myovirus	$(1.43 \pm 0.40) \times 10^8$	1	40.9	2	-0.0262 ± 0.2971	1	1	0.064 ± 0.005	Effective
ϕ385	Podovirus	$(1.53 \pm 0.15) \times 10^6$	3	36.4	2	-0.0510 ± 0.0182	1	1	0.106 ± 0.041	Insufficient
ϕR34	Podovirus	$(2.73 \pm 0.21) \times 10^8$	1	36.4	2	-0.0753 ± 0.2588	1	1	0.094 ± 0.001	Effective
ϕRB64	Myovirus	$(2.13 \pm 0.15) \times 10^8$	1	40.9	2	-0.0459 ± 0.2301	1	1	0.061 ± 0.008	Effective
ϕJB96	Myovirus	$(1.40 \pm 0.17) \times 10^8$	1	40.9	2	-0.1100 ± 0.2474	1	1	0.063 ± 0.005	Effective
ϕ4C01	Myovirus	$(3.27 \pm 0.25) \times 10^6$	3	100.0	1	0.2498 ± 0.3033	3	1	0.226 ± 0.163	Insufficient
ϕR35	Podovirus	$(1.27 \pm 0.25) \times 10^8$	1	36.4	2	-0.0232 ± 0.1042	1	1	0.095 ± 0.006	Effective
ϕRB66	Podovirus	$(2.37 \pm 0.15) \times 10^6$	3	36.4	2	0.0660 ± 0.0837	2	1	0.125 ± 0.057	Insufficient

ϕRB97	Podovirus	(1.47 ± 0.15) × 10^6	3	36.4	2	0.1046 ± 0.1581	3	1	0.240 ± 0.085	Insufficient
ϕ4C08	Myovirus	(6.57 ± 0.21) × 10^7	2	100.0	1	0.3825 ± 0.1055	4	1	0.059 ± 0.006	Effective
ϕR36	Podovirus	(3.37 ± 0.21) × 10^8	1	36.4	2	-0.2242 ± 0.0179	1	1	0.091 ± 0.003	Effective
ϕRB67	Podovirus	(2.77 ± 0.06) × 10^7	2	36.4	2	-0.0071 ± 0.1772	1	1	0.095 ± 0.007	Effective
ϕJB98	Podovirus	(3.20 ± 0.26) × 10^7	2	81.8	1	-0.2180 ± 0.0927	1	1	0.064 ± 0.009	Effective
ϕ5C05	Podovirus	(2.27 ± 0.38) × 10^8	1	36.4	2	0.0453 ± 0.0620	2	2	0.102 ± 0.021	Insufficient
ϕR37	Podovirus	(2.50 ± 0.20) × 10^8	1	36.4	2	-0.2474 ± 0.0354	1	1	0.104 ± 0.024	Insufficient
ϕRB70	Myovirus	(3.67 ± 0.15) × 10^6	3	100.0	1	0.5280 ± 0.1180	4	1	0.124 ± 0.060	Insufficient
ϕRB99	Podovirus	(2.17 ± 0.15) × 10^8	1	36.4	2	-0.1974 ± 0.0384	1	1	0.110 ± 0.018	Insufficient
ϕYB12	Podovirus	(3.20 ± 0.26) × 10^6	3	36.4	2	0.1049 ± 0.0118	3	2	0.096 ± 0.007	Effective
ϕR38	Podovirus	(5.47 ± 0.06) × 10^7	2	36.4	2	-0.0111 ± 0.0411	1	1	0.095 ± 0.017	Effective
ϕRB71	Podovirus	(2.20 ± 0.20) × 10^6	3	36.4	2	0.0537 ± 0.0564	2	1	0.142 ± 0.007	Insufficient
ϕRB101	Podovirus	(3.40 ± 0.17) × 10^6	3	36.4	2	0.4418 ± 0.0586	4	1	0.139 ± 0.055	Insufficient
ϕYB16	Podovirus	(5.57 ± 0.21) × 10^6	3	36.4	2	0.0662 ± 0.0229	2	2	0.096 ± 0.009	Effective
ϕR39	Podovirus	(1.37 ± 0.21) × 10^6	3	36.4	2	-0.0671 ± 0.0301	1	1	0.160 ± 0.080	Insufficient
ϕRB73	Podovirus	(1.37 ± 0.15) × 10^6	3	36.4	2	-0.0059 ± 0.0567	1	1	0.152 ± 0.046	Insufficient
ϕJB104	Podovirus	(1.57 ± 0.12) × 10^6	3	36.4	2	0.1884 ± 0.1401	3	2	0.249 ± 0.100	Insufficient
ϕR18	Podovirus	(2.20 ± 0.26) × 10^8	1	36.4	2	-0.2415 ± 0.0248	1	1	0.060 ± 0.004	Effective
ϕYB47	Myovirus	(1.67 ± 0.15) × 10^9	1	40.9	2	0.1416 ± 0.2802	3	1	0.067 ± 0.007	Effective
ϕRB74	Podovirus	(3.10 ± 0.17) × 10^6	3	36.4	2	0.3281 ± 0.0780	4	1	0.186 ± 0.074	Insufficient
ϕJB105	Podovirus	(1.73 ± 0.21) × 10^6	3	36.4	2	0.1087 ± 0.0803	3	2	0.248 ± 0.054	Insufficient
ϕR21	Podovirus	(1.53 ± 0.15) × 10^8	1	36.4	2	-0.2270 ± 0.0282	1	1	0.100 ± 0.029	Insufficient
ϕR48	Myovirus	(1.80 ± 0.10) × 10^8	1	77.3	1	0.0142 ± 0.3470	2	1	0.101 ± 0.014	Insufficient
ϕRB75	Podovirus	(3.23 ± 0.21) × 10^7	2	36.4	2	-0.0702 ± 0.1113	1	1	0.093 ± 0.003	Effective
ϕJB106	Podovirus	(1.53 ± 0.25) × 10^6	3	36.4	2	0.0626 ± 0.0514	2	2	0.260 ± 0.076	Insufficient
ϕR24	Podovirus	(2.17 ± 0.23) × 10^6	3	36.4	2	-0.0291 ± 0.0645	1	1	0.098 ± 0.011	Effective
ϕR49	Myovirus	(7.60 ± 0.26) × 10^6	3	95.5	1	0.2227 ± 0.3040	3	1	0.117 ± 0.063	Insufficient
ϕRB76	Podovirus	(1.23 ± 0.21) × 10^8	1	36.4	2	-0.0053 ± 0.1599	1	1	0.091 ± 0.002	Effective
ϕJB107	Podovirus	(2.40 ± 0.40) × 10^6	3	36.4	2	0.0919 ± 0.0882	2	2	0.248 ± 0.103	Insufficient
ϕEB108	Podovirus	(6.47 ± 0.15) × 10^7	2	36.4	2	-0.2041 ± 0.0520	1	1	0.106 ± 0.016	Insufficient

Note: values are the mean ± standard deviation of three independent replicates (n = 3). Levels are those assigned from the original determinations and are reproduced here unchanged. Coverage rate is the proportion of the 22 phage-resistant mutants infected by each secondary phage and was determined once; plaque morphology was graded once. Outcome refers to inhibition of CRAB 43895 by the ϕ8 cocktail at 96 h (effective, $OD_{600} < 0.1$). The complete replicate-level dataset is available from the corresponding author on request.

Table S5. Complete output of the multivariable logistic regression model.

Term	β	SE	z	*p*-value	Odds ratio	95% CI
Intercept	-0.614	1.404	-0.44	0.662	—	—
RBG, level 3	-0.630	1.318	-0.48	0.633	0.53	0.04–7.05
RBG, level 2	-1.343	1.347	-1.00	0.318	0.26	0.02–3.65
RBG, level 1	-0.077	1.219	-0.06	0.950	0.93	0.09–10.09
Lytic capability, level 2	2.207	0.910	2.43	0.015	9.09	1.53–54.06
Lytic capability, level 1	2.463	0.862	2.86	0.004	11.74	2.17–63.56
Plaque morphology, level 1	-0.349	1.144	-0.31	0.760	0.71	0.08–6.64
Coverage rate, level 1	0.370	0.967	0.38	0.702	1.45	0.22–9.64

Note: n = 65 with 36 events and 7 estimated parameters. Reference categories are RBG level 4, lytic capability level 3, coverage rate level 2 and plaque morphology level 2. The odds ratios and confidence intervals are those reported in Table 3. Log-likelihood = -33.90; AIC = 83.8; likelihood-ratio $\chi^2 = 21.55$ ($p = 0.0030$); McFadden pseudo-$R^2 = 0.241$.

Table S6. Sensitivity of the logistic regression model to the definition of effective inhibition.

96-h OD_{600} threshold	Effective / insufficient	Lytic capability	RBG	Coverage rate	Plaque morphology
0.08	19 / 46	0.013*	0.723	0.382	0.703
0.09	21 / 44	0.017*	0.679	0.491	0.384
0.10	36 / 29	0.009*	0.584	0.764	0.768
0.11	43 / 22	<0.001*	0.685	0.461	0.474
0.12	46 / 19	<0.001*	0.666	0.116	0.602
0.15	55 / 10	0.121	0.418	0.453	0.451
0.20	59 / 6	0.575	0.139	0.874	0.216

Note: the outcome (effective inhibition) was redefined at each 96-h OD_{600} threshold and the multivariable model refitted by Firth penalized likelihood, each indicator being tested as a whole rather than level by level (Section 4.11). *$p < 0.05$.

Table S7. Confirmatory experiment: 96-h OD_{600} and viable counts for six representative secondary phages against the clinical strain CRAB 43895.

Phage	Treatment	96-h OD_{600}, mean ± SD	Outcome	CFU/mL at 0 h	CFU/mL at 24 h	CFU/mL at 96 h
ϕR18	Cocktail (total nominal MOI 10)	0.066 ± 0.004	Effective	$(4.50 \pm 2.29) \times 10^6$	< 500	< 500
ϕR18	Cocktail (total nominal MOI 5; original condition)	0.070 ± 0.004	Effective	$(3.67 \pm 1.89) \times 10^6$	< 500	< 500
ϕR18	Secondary phage alone (nominal MOI 10)	0.066 ± 0.004	Effective	$(4.33 \pm 0.76) \times 10^6$	< 500	< 500
ϕR32	Cocktail (total nominal MOI 10)	0.064 ± 0.001	Effective	$(4.33 \pm 2.52) \times 10^6$	< 500	< 500
ϕR32	Cocktail (total nominal MOI 5; original condition)	0.067 ± 0.004	Effective	$(2.50 \pm 0.00) \times 10^6$	< 500	< 500
ϕR32	Secondary phage alone (nominal MOI 10)	0.064 ± 0.001	Effective	$(3.17 \pm 0.29) \times 10^6$	< 500	< 500
ϕJB98	Cocktail (total nominal MOI 10)	0.067 ± 0.003	Effective	$(4.00 \pm 1.73) \times 10^6$	< 500	< 500
ϕJB98	Cocktail (total nominal MOI 5; original condition)	0.064 ± 0.005	Effective	$(4.33 \pm 0.58) \times 10^6$	< 500	< 500
ϕJB98	Secondary phage alone (nominal MOI 10)	0.062 ± 0.001	Effective	$(3.67 \pm 0.76) \times 10^6$	< 500	< 500
ϕ4C01	Cocktail (total nominal MOI 10)	0.230 ± 0.001	Insufficient	$(4.00 \pm 1.00) \times 10^6$	$(2.83 \pm 1.61) \times 10^6$	$(2.67 \pm 0.76) \times 10^7$
ϕ4C01	Cocktail (total nominal MOI 5; original condition)	0.235 ± 0.051	Insufficient	$(3.33 \pm 2.36) \times 10^6$	$(2.33 \pm 0.29) \times 10^6$	$(2.83 \pm 1.44) \times 10^7$
ϕ4C01	Secondary phage alone (nominal MOI 10)	1.062 ± 0.086	Insufficient	$(5.17 \pm 1.26) \times 10^6$	$(3.83 \pm 1.26) \times 10^8$	$(1.33 \pm 0.29) \times 10^9$
ϕJB53	Cocktail (total nominal MOI 10)	0.079 ± 0.012	Effective	$(3.50 \pm 1.32) \times 10^6$	$(3.33 \pm 0.58) \times 10^6$	< 500
ϕJB53	Cocktail (total nominal MOI 5; original condition)	0.079 ± 0.012	Effective	$(2.83 \pm 1.26) \times 10^6$	$(1.47 \pm 0.90) \times 10^6$	< 500
ϕJB53	Secondary phage alone (nominal MOI 10)	1.072 ± 0.068	Insufficient	$(5.00 \pm 1.80) \times 10^6$	$(2.33 \pm 1.04) \times 10^8$	$(8.00 \pm 1.00) \times 10^8$
ϕJB106	Cocktail (total nominal MOI 10)	0.135 ± 0.095	Insufficient	$(4.67 \pm 1.53) \times 10^6$	$(3.14 \pm 5.07) \times 10^6$	$(3.50 \pm 2.60) \times 10^6$

ϕJB106	Cocktail (total nominal MOI 5; original condition)	0.260 ± 0.106	Insufficient	$(5.00 \pm 0.50) \times 10^{6}$	$(2.00 \pm 1.00) \times 10^{7}$	$(2.83 \pm 1.76) \times 10^{7}$
ϕJB106	Secondary phage alone (nominal MOI 10)	0.924 ± 0.163	Insufficient	$(3.67 \pm 1.15) \times 10^{6}$	$(1.50 \pm 0.87) \times 10^{8}$	$(8.33 \pm 1.04) \times 10^{8}$
—	ϕ8 alone (nominal MOI 10)	0.544 ± 0.101	Insufficient	$(3.00 \pm 1.00) \times 10^{6}$	$(2.00 \pm 1.32) \times 10^{6}$	$(4.17 \pm 0.76) \times 10^{8}$
—	Untreated bacterial control	1.263 ± 0.068	Insufficient	$(4.00 \pm 1.00) \times 10^{6}$	$(1.33 \pm 0.58) \times 10^{8}$	$(2.00 \pm 1.32) \times 10^{9}$
—	LB control	0.049 ± 0.001	—	< 500	< 500	< 500

Note: Values are the mean ± SD of three independent wells (n = 3). For viable counts, three technical spot counts were averaged to obtain one value per well. The nominal inputs were 5×10^{4} CFU per well and either 5.0×10^{5} PFU (total nominal MOI 10) or 2.5×10^{5} PFU (total nominal MOI 5). Each phage contributed half of the total PFU in the cocktail treatments. Treatments with a mean 96-h OD_{600} below 0.1 were classified as effective. Viable counts were reported as <500 CFU/mL when no colonies were detected in three undiluted 2-µL spots. MOIs were calculated from the OD-estimated bacterial inoculum and prepared phage concentrations; the 0-h viable counts were measured independently after mixing.
